# High functional allelic diversity and copy number in both MHC classes in the common buzzard

**DOI:** 10.1186/s12862-023-02135-9

**Published:** 2023-06-24

**Authors:** Jamie Winternitz, Nayden Chakarov, Tony Rinaud, Meinolf Ottensmann, Oliver Krüger

**Affiliations:** Department of Animal Behaviour, Bielefeld University, Morgenbreede 45, 33615 Bielefeld, Germany

**Keywords:** Major histocompatibility complex, *Buteo buteo*, Birds of prey, Long-read sequencing, High-throughput sequencing, RNAseq, Genotyping, Characterization, Selection, Copy number variation, Peptide-binding groove, Peptide binding residues, Trans-species polymorphism, Nonclassical MHC

## Abstract

**Background:**

The major histocompatibility complex (MHC), which encodes molecules that recognize various pathogens and parasites and initiates the adaptive immune response in vertebrates, is renowned for its exceptional polymorphism and is a model of adaptive gene evolution. In birds, the number of MHC genes and sequence diversity varies greatly among taxa, believed due to evolutionary history and differential selection pressures. Earlier characterization studies and recent comparative studies suggest that non-passerine species have relatively few MHC gene copies compared to passerines. Additionally, comparative studies that have looked at partial MHC sequences have speculated that non-passerines have opposite patterns of selection on MHC class I (MHC-I) and class II (MHC-II) loci than passerines: namely, greater sequence diversity and signals of selection on MHC-II than MHC-I. However, new sequencing technology is revealing much greater MHC variation than previously expected while also facilitating full sequence variant detection directly from genomic data. Our study aims to take advantage of high-throughput sequencing methods to fully characterize both classes and domains of MHC of a non-passerine bird of prey, the common buzzard (*Buteo buteo*), to test predictions of MHC variation and differential selection on MHC classes.

**Results:**

Using genetic, genomic, and transcriptomic high-throughput sequencing data, we established common buzzards have at least three loci that produce functional alleles at both MHC classes. In total, we characterize 91 alleles from 113 common buzzard chicks for MHC-I exon 3 and 41 alleles from 125 chicks for MHC-IIB exon 2. Among these alleles, we found greater sequence polymorphism and stronger diversifying selection at MHC-IIB exon 2 than MHC-I exon 3, suggesting differential selection pressures on MHC classes. However, upon further investigation of the entire peptide-binding groove by including genomic data from MHC-I exon 2 and MHC-IIA exon 2, this turned out to be false. MHC-I exon 2 was as polymorphic as MHC-IIB exon 2 and MHC-IIA exon 2 was essentially invariant. Thus, comparisons between MHC-I and MHC-II that included both domains of the peptide-binding groove showed no differences in polymorphism nor diversifying selection between the classes. Nevertheless, selection analysis indicates balancing selection has been acting on common buzzard MHC and phylogenetic inference revealed that trans-species polymorphism is present between common buzzards and species separated for over 33 million years for class I and class II.

**Conclusions:**

We characterize and confirm the functionality of unexpectedly high copy number and allelic diversity in both MHC classes of a bird of prey. While balancing selection is acting on both classes, there is no evidence of differential selection pressure on MHC classes in common buzzards and this result may hold more generally once more data for understudied MHC exons becomes available.

**Supplementary Information:**

The online version contains supplementary material available at 10.1186/s12862-023-02135-9.

## Background

Immune genes include some of the most polymorphic genes in the genomes of vertebrates and invertebrates [1] because of their role in protection against numerous parasites [1–3]. For vertebrates, the major histocompatibility complex (MHC), encoding molecules responsible for recognizing antigens and presenting them to stimulate important arms of the adaptive immune system, stands out for its unparalleled diversity. For example, hundreds and in some cases thousands of allelic variants have been identified in natural populations of reptiles [4], birds [5, 6], bats [7], and humans [8]. This variation is believed to be generated via mutation, gene duplication, and gene conversion [9, 10] and maintained by constant selection pressure from various co-evolving macro and microparasites [11].

The extraordinary polymorphism of classical MHC genes derives from their function. Classical MHC genes encode cell surface receptors that present self and non-self peptides to lymphocytes responsible for effective immune response [12, 13]. “Classical MHC molecules” have three essential properties: high polymorphism, high ubiquitous expression, and peptide presentation. In contrast, “nonclassical MHC molecules” are related to classical MHC molecules and may be structurally similar, but they lack one or more of the three essential properties of classical MHC molecules and have different functions that may or may not be immune related [14]. For example, the nonclassical human MHC (human leucocyte antigen, HLA) HLA-G encodes molecules that bind peptides [15], but has 110–150 times fewer protein alleles than classical HLA-A, -B, and -C [16], is predominantly expressed in the placenta during pregnancy, and has a role in immune suppression [17]. Because we are interested in MHC polymorphism related to pathogen pressure, we will be focusing on classical MHC molecules. The classical MHC exists as a multigene family with two main subclasses, class I (MHC-I) and class II (MHC-II). Classical MHC-I molecules are expressed on nearly all nucleated cells. They present peptides originating from intracellular sources (e.g. self-derived peptides and peptides originating from viruses or other pathogens that have entered the cell) to cytotoxic T cells which, once activated, can initiate the death of the cell. Classical MHC-II molecules are expressed constitutively by professional antigen-presenting cells (e.g. macrophages, B cells and dendritic cells, among others), and present peptides originating from exogenous sources (e.g. derived from bacteria or parasite particles that have been ingested by the cell) [18]. MHC molecules bind pathogen antigens at the peptide-binding groove, which encompasses the peptide binding amino acid residues (PBR). The peptide-binding groove is formed by two molecular domains: α1 and α2 in MHC class I (coded by exon 2 and exon 3 for MHC class I genes) [19] and α1 and β1 in MHC class II (coded by exon 2 for MHC class II A and B genes) [20].

Evidence of the adaptive nature of high sequence polymorphism is that higher rates of amino acid changing substitutions occur at PBR of the MHC molecule, the sites interacting directly with antigens [19, 20]. Molecules coded by different MHC alleles differ in their binding affinities for specific pathogen peptides [21], so multiple alleles are required to confer resistance to different pathogen genotypes and species. Thus, on average, heterozygous individuals should exhibit greater pathogen recognition than homozygous individuals [22], though not all alleles are equal and can be ‘generalists’, binding a wider range of peptide motifs, and ‘specialists’, binding a small number of very similar peptides [23, 24]. Similarly, populations exposed to many different pathogens should have greater numbers of alleles and ‘generalist’ alleles than populations with lower pathogen richness [24–26]. The emerging view is that pathogen-mediated balancing selection plays a major role maintaining MHC population allelic diversity [11] as well as preserving adaptive allelic diversity across speciation events, or trans-species polymorphism [27, 28].

Comparative studies of mammals [29] and birds [30, 31] have shown great variation in allelic diversity and locus copy number across taxa. In birds, low copy number is believed to be the ancestral state and common in non-passerines [31]. In contrast, passerines have MHC locus variation ranging from few to dozens of copies [30]. For birds, there is accumulating evidence that life-history strategies that increase exposure to pathogen pressure are responsible for higher loci copy numbers [32] and stronger signatures of diversifying selection [33]. Recent studies have also shown that patterns of selection and sequence polymorphism in birds differ between MHC-I and MHC-II and speculate that these differences are also driven by relative selective pressure imposed by microparasites and macroparasites, respectively [34–36]. However, all the studies that support this hypothesis have only focused on MHC-I exon 3 and MHC-IIB exon 2, encoding only half of the class I and class II peptide-binding grooves. Most MHC studies have focused on these exons that are believed by some researchers to be responsible for the majority of functional polymorphism and the main targets of pathogen-mediated selection in birds [37–39]. Indeed, these exons were our original focus as well. Data on the corresponding exons MHC-I exon 2 and MHC-IIA exon 2 encoding peptide-binding domains is extremely limited and so the extent of polymorphism and selection at these understudied exons is unclear. Nevertheless, studies that genotype populations at both MHC classes and both domains (i.e. MHC-I exon 2 and 3, MHC-IIA exon 2 and MHCII-B exon 2) of the peptide-binding groove are required to test if patterns of selection and sequence polymorphism differ between MHC-I and MHC-II in birds.

Birds of prey make useful non-model systems to investigate MHC evolution because thus far, the evidence gathered shows that they have few MHC loci [e.g., 1, 2, 40, 41]. However, comparative evidence suggests that residual lifespan correlates positively with higher number of loci, suggestive of higher parasite exposure in longer-lived species [34], like birds of prey. Additionally, birds of prey have open nests with high site fidelity which leads to predictable exposure to parasites and vectors of pathogens [40–42]. Thus, the MHC in birds of prey is expected to be under strong pathogen-mediated selection. Common buzzards are useful to study because they are widespread and have been longitudinally studied for over 30 years in the Eastern Westphalia area with detailed records of genetic, recruitment, and fitness data [43, 44].

Taking advantage of the exciting promise of high-throughput sequencing methods for increasing understanding of avian MHC evolution [45], our study intends to: (i) characterize both MHC-I exon 3 and MHC-IIB exon 2 polymorphism and genomic structure in a common buzzard population, (ii) test for signatures of historic selection acting on peptide binding sites, suggesting that parasite-mediated selection is acting to maintain diversity at these loci, (iii) conduct phylogenetic analysis among related avian species to test for evidence of trans-species polymorphism. Motivated by reviewer comments, we also wanted to make full use of our long-read data and (iv) identify the other domains of class I and II peptide-binding grooves, MHC-I exon 2 and MHC-IIA exon 2, to see the complete picture of peptide binding region variation within and between the MHC classes.

## Results

### MHC-I exon 3 genotyping

A total of 91 putative sequence variants (hereafter referred to as ‘alleles’) of 262 bp were identified for MHC-I exon 3: Bubute class I-N*01–91 (Genbank accession #s: OL311188‒ OL311278). MHC nomenclature from this study takes the first 2 characters of the genus-species name (Bubu) and since this was already taken for multiple species (e.g., *Bufo bufo*, *Bubalus bubalis*, *Bubo bubo*), we use the next 2 characters of the species name until a unique name was generated (following the recommendations of [46]). Additionally, as class I sequences cannot be assigned to individual loci at present, we assign the allelic series under the prefix *N* until it can be replaced by a number (following recommendations of [47]). All alleles had blast hits (E value < 1e^−5^) with avian MHC-I exon 3 loci between 90.8% to 98.9% identity. These alleles translated (on reading frame 2) without stop codons and showed high conservation with residues known to be structurally important features of classical MHC-I loci (Additional file Figure S1).

To assess if our alleles could potentially bind peptides and fulfill the peptide presentation requirement of “classical MHC” status, we looked at the five positions in exon 3 (T143, K146, W147,Y159, Y171, using the human sequence HLA-A2:01 as reference) of the eight highly conserved positions of class I molecules that bind the N- and C-terminal ends of peptides [48]. All sites were perfectly conserved except one: K146 (Additional file Figure S1 and S3). In this case, ten out of 91 sequences had an arginine (R) instead of lysine (K) at this site. However, both R and K have basic side chains so this substitution might not interfere with peptide binding. In support of the substitution's preserved functionality, multiple avian and anuran species have the residue R146 instead of K146 at presumably classical MHC-I exon 3 sequences based on expression and polymorphism patterns [49, 50]. Thus, based on perfect conservation at most peptide-anchoring sites and presumed conservation of function at K/R146 that is shared among non-mammals, we conclude our molecules can bind peptides, and thus satisfy one of the three essential properties that define classical MHC molecules.

We sequenced 130 samples, 17 of which had fewer than 100 reads required for genotyping so were removed from the final list of genotyped MHC-I exon 3 samples. In total, we had MHC-I exon 3 genotypes for 113 individuals, 65 of which (57.5%) had technical replicates with an average reproducibility of alleles of 96.2%. The average number of reads ± [SE] per sample was 1940 ± 204. There was no statistically significant linear relationship between allele count and read depth (R^2^ = 0.003, F(1,111) = 0.277, *p* = 0.600, Additional Figure S2). The range of alleles per genotype was 1 to 8, and the average was 4.81. Five samples (IDs 102, 103, 104, 105, and 106) had the same single MHC-I exon 3 allele each (Bubute class I-N*01). The data of MHC-I exon 3 alleles for each individual, with read numbers, is available on the figshare repository (DOI: 10.6084/m9.figshare.16885255).

Three individuals had 7–8 alleles (ID-65, ID-93, ID-94), more than the 6 alleles expected based on evidence of three MHC-I exon 3 loci from genomic HiFi data of 4 individuals. The individuals with 7–8 alleles had technical replicates sharing 5, 2, and 3 alleles, respectively, so the replicates could not confirm that more than 6 alleles were present (assuming 3 loci max). However, further inspection indicated that the genotypes were accurate as all had alleles found in more than one individual, that were more than 7 bp different from other alleles within the amplicon, and were at relatively high frequency (≥ 5%). Individual ID-94 had had 7 alleles, 5 of which were shared with a genotyped sibling in the dataset (ID-95). The two alleles not shared by its sibling (Bubute class I-N*40 and N*61) are also only present in the individual with 8 alleles (ID-93), and Bubute class I-N*40 was found to be expressed. Thus, while the 7–8 allele genotypes could represent random contamination that was not recovered in negative controls or PCR/sequencing artifacts, they more likely indicate that copy number variation (CNV) for a fourth MHC-I exon 3 locus may be present within this population, which is corroborated by our long-read genomic data (see section ‘Copy number and both peptide binding region domains determined using genomic long-read data’). We attempted to assign alleles to loci directly from the phenotypes using MHC-Typer V1.1, a maximum-likelihood method of haplotype reconstruction that takes into account null alleles or CNV, deviation from Hardy–Weinberg Equilibrium, and sharing of identical alleles between loci [51]. However, multiple independent runs did not converge at the same optimal Bayesian information criterion (BIC) using the suggested two-step method with the settings as follows: Step 1: random initial solution for #loci = 4, number of repeats = 10, chain length = 500, initial temperature = 0.01, final temperature = 0.00001, anneal coefficient = 0.99, max iteration = 30, min freq. diff = 0.002, penalty of missing = -2, penalty of mismatch = -80, taboo coef. = 1; Step 2: initial solution for loci = solution from best run of Step 1, number of repeats = 300, initial temperature = 0.0001, final temperature = 0.000001, consider null alleles, consider deviation from HWE, initial null allele freq. = 0.05, penalty of missing = 0, penalty of mismatch = -1000, taboo coef. = 1.00001. The failure to reach a correct assignment is likely due to the high complexity of our system (≥ 3 loci with 91 alleles), for which we would need a much greater sample size to reach a correct assignment rate of 80–95% (e.g., 400 to 1300 genotyped individuals [51]).

### MHC-IIB exon 2 genotyping

A total of 41 putative alleles of 258 bp were identified for MHC-IIB exon 2: Bubute-DRB*01‒*40 (Genbank accession #s: OL311279‒OL311318) and the previously published Butbut-DAB*01 (accession # KJ162430). As there were multiple names given to previously described *Buteo buteo* DRB/DAB alleles (e.g., *Butbu-DRB* from [52], *Buteo-DRB* from [53], and *Butbut-DAB* from [54], we decided to be consistent and follow the new nomenclature recommendations of [46]. All alleles had blast hits (E value < 1e^−5^) with avian MHC-II exon 2 loci between 90.3% to 100% identity (for Butbut-DAB*01). Alleles translated (on reading frame 3) without stop codons and showed high conservation with residues known to be structurally important features of classical MHC-II loci (Additional file Figure S3). Six putative alleles (Bubute-DRB*05, Bubute-DRB*06, Bubute-DRB*08, Bubute-DRB*13, Bubute-DRB*16, Bubute-DRB*29) had a 3 bp deletion that translated to position 78 based on the complete HLA-DRB1 exon 2 as a reference. This deletion has frequently been observed in non-passerine and passerine families (21 species total) across phylogenetically distinct clades [34].

We assessed the peptide presentation requirement of “classical molecules” by looking at the three invariant positions of class IIB molecules (W61, H81, N82, using the human sequence HLA-DRB1:0101 as reference) that bind the main chain of the peptide [48]. All sites were perfectly conserved. Therefore, we conclude our molecules can bind peptides, and thus satisfy one of the three essential properties that define classical MHC molecules.

We genotyped 125 individuals, 81 of which (64.8%) had technical replicates with an average reproducibility of 98.1%. The average number of reads ± [SE] per sample was 1475 ± 121. A linear regression showed no statistically significant relationship between allele count and read depth (R^2^ = 0.018, F(1,123) = 2.226, *p* = 0.138, Additional file Figure S4). The range of alleles per genotype was 2 to 5, and the average was 3.65. The data of MHC-IIB exon 2 alleles for each individual, with read numbers, is available on the figshare repository (DOI: 10.6084/m9.figshare.16885255). Ten individuals with an average number of reads ± [SE] of 1509 ± 565 had only two alleles each, which suggests either CNV, poor PCR primer amplification for certain loci, or that alleles are shared across loci. As with MHC-I exon 3, maximum-likelihood haplotype reconstruction using MHC-Typer V1.1 did not achieve convergence following the same two-step procedure as above but also allowing loci to share identical alleles, and thus more intensive haplotyping measures (e.g., high coverage long-read sequencing, amplification with locus-specific primers) would be required to confirm either CNV or allele-sharing across multiple loci.

### Copy number and both peptide binding region domains determined using genomic long-read data

Using buzzard hybrid PacBio Hifi-HiC scaffold assemblies (see Methods for details) allowed us to confirm that both MHC class I exon 3 and class II DRB exon 2 are present in at least three locations in *Buteo buteo* chromosome 29, with potential CNV for fourth locus at class II (Additional file Figure S5). While this is the first study that has characterized MHC-I in common buzzards, two other studies have characterized common buzzard MHC-IIB. Sampling single individuals for PCR and cloning and inferring loci number from maximum number of sequences amplified, they estimated MHC-IIB loci number ranges from 1 to greater than 1 copy [52, 54]. The discrepancy between previous estimates and our identification of at least three loci present at MHC-IIB could be explained if CNV is present, if alleles are shared among loci, or if cloning and sequencing underestimated the true number of alleles present.

Genomic long-read HiFi data also allowed us to retrieve two additional exons encoding the other half of the peptide-binding groove of MHC-I and MHC-II, specifically MHC-I exon 2 and MHC-IIA exon 2. It was necessary to restrict our analyses to contig data that was haplotype-aware and thus did not collapse polymorphisms from different alleles together. While haplotype-aware contig data was only available for four individuals and coverage across the MHC region was incomplete, we did identify 10 alleles (out of 14 sequences retrieved) of 264 bp for MHC-I exon 2: Bubute class I N*01‒*10 *exon 2* (accession #s: OP503112‒ OP503121) and 3 alleles (out of 12 sequences retrieved) of 258 bp for MHC-IIA exon 2: Bubute-DRA*01‒*03 (accession #s: OP490257‒ OP490259). Bubute class I-N*07 exon 2 was manually edited to remove frameshift insertions (extra C between sites 55 and 56 and extra A between sites 251 and 252) caused by the common problem of tandem base pair repeats during assembly [55]. All alleles had blast hits (E value < 1e^−83^) with avian MHC-I exon 2 loci between 87.2% to 98.6% identity and blast hits (E value < 1e^−113^) with MHC-IIA exon 2 loci between 96.8% and 97.6% identity. Full- or nearly full-length MHC-I and II alleles and coding sequences from haplotype-aware contig data were predicted and edited based on blast alignments and RNAseq evidence and can be found at the GenBank accession numbers OL311287, OL311290, OL311292, OL311294, OL311304, OL311305, OP490259, OQ414190-OQ414202, OQ428163-OQ428174.

### Confirming allele expression with RNA-seq data

**Fig. 1:**
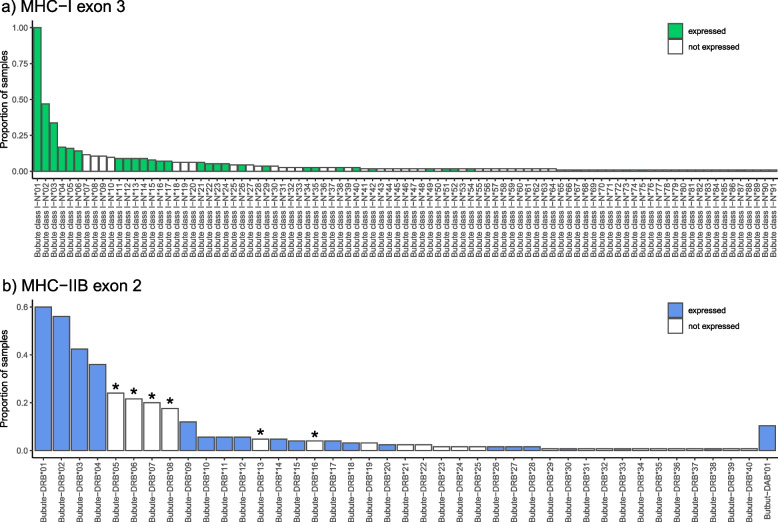
MHC allele frequency and expression patterns in common buzzards. The proportion of individuals carrying each allele are shown for **a**) MHC-I exon 3 and for **b**) MHC-IIB exon 2. Colored bars indicate alleles that were shown to be expressed. Stars in **b**) indicate MHC-IIB alleles that have a 3 bp deletion

Genomic RNA was sequenced from whole blood for another study from 81 individuals, 37 of whom also had MHC genotype data. For MHC-I, 35% of alleles were expressed (32/91 alleles), and common alleles were more likely to be expressed (Fig. 1a). One expressed allele, Bubute class I-N*01, was found in every MHC genotyped individual. Since MHC expression data was not the primary objective of the RNAseq experiments, coverage of alleles was relatively low (avg read number ± SE: 254.27 ± 27.12). For example, 56% of MHC-I exon 3 alleles present across the 37 individuals with both RNAseq and MHC genotype data were recorded as expressed (22/39 alleles). For MHC-IIB exon 2, 49% of alleles were expressed (20/41 alleles), with 36% of alleles expressed that were present in individuals with both RNAseq and MHC genotype data (9/25 alleles). As with MHC-I exon 3, common alleles were more likely to be expressed (Fig. 1b). Interestingly, the six relatively common MHC-IIB exon 2 alleles with a 3 bp deletion at AA position 78 (allele [frequency]: Bubute-DRB*05 [0.24], Bubute-DRB*06 [0.22], Bubute-DRB*08 [0.18], Bubute-DRB*13 [0.05], Bubute-DRB*16 [0.04], Bubute-DRB*29 [0.01]) were not found to be expressed.

Besides confirming allele expression for MHC-I exon 3 and MHC-IIB exon 2, we were also able to confirm expression of 6 out of 10 MHC-I exon 2 sequences and all three MHC-IIA exon 2 sequences. The expression of these sequences and MHC-I exon 3 and MHC-IIB exon 2 sequences from haplotype-aware contigs confirms that all three loci at both classes are expressed (Additional file Table S4).

### Sequence polymorphism

Although MHC-I exon 3 had higher allelic diversity than MHC-IIB exon 2 (91 vs. 41 alleles), sequence diversity at the nucleotide and amino acid level was greater for MHC-IIB exon 2. MHC class IIB exon 2 had a higher number of polymorphic sites, mutations, average number of nucleotide differences, nucleotide diversity, and number of amino acid differences per site than MHC class I exon 3 (Table 1). However, including the long-read derived MHC-I exon 2 and MHC-IIA exon 2 data, it appears that MHC class I and II binding grooves have similar sequence polymorphism. In fact, amino acid polymorphism at MHC-I exon 2 is even higher than at exon 3, on par with polymorphism at MHC-IIB exon 2 (Table 1). In contrast, MHC-IIA exon 2 is almost monomorphic, with only three bp and three amino acid differences detected out of 12 sequences recovered (Additional file Figure S6b).

**Table 1:** Sequence polymorphism for MHC class I and class II of common buzzards (*Buteo buteo*)

MHC class	Exon	N	n. nuc	n. alleles	S	Eta	k	Pi	AA p-distance
MHC-I	2	4	264	10	58	66	24.844	0.094	0.173
MHC-I	3	113	262	91	58	67	15.121	0.058	0.105
MHC-IIA	2	4	258	3	3	3	2.000	0.008	0.016
MHC-IIB	2	125	258	41	77	100	26.574	0.104	0.178

*N* sample size, *n. nuc* number of nucleotides, *n. alleles* number of alleles, *S* number of polymorphic sites, Eta: total number of mutations, k: average number of nucleotide differences, Pi: nucleotide diversity, AA p-distance: number of amino acid differences per site

The pairwise identity for MHC-I exon 2 was 90.6% and for exon 3 was 94.2%, representing 9.4% and 5.8% divergence, respectively. Pairwise identify for MHC-IIA and MHC-IIB exon 2 was 99.2% and 89.3%, representing 0.8% and 10.7% divergence, respectively. Thus, MHC-I and MHC-IIB exon 2 alleles met the minimum sequence divergence of 5% required for adequate power by most recombination detection methods [56], and so we proceeded with recombination analyses.

### Recombination

Recombination is frequent in MHC genes, including avian MHC genes [34], and may lead to overestimation of the number of positively selected sites. Therefore, recombination was tested prior to analyses using GARD [57] and RPD4 v.4.101 software [58]. One recombination breakpoint at bp 104 was detected for MHC-I exon 2 using GARD and 105 using RDP4, so downstream selection analysis was conducted on partitioned data exported from GARD. For MHC-I exon 3, there were no recombination events detected by either method that met our threshold of validation. For MHC-IIB exon 2, no recombination events were detected by GARD but RDP4 identified 24 (of 41) alleles as recombination products from three recombination events (Additional file Table S2). These recombinants represented 59% of the MHC-IIB exon 2 alleles and had breakpoints (128–257, 182–254, 53–185) identified by three or more methods. Thus, downstream selection analysis was conducted on full sequence alignments and on alignments with recombinant segments removed from sequences.

### Inference of selection

**Fig. 2:**
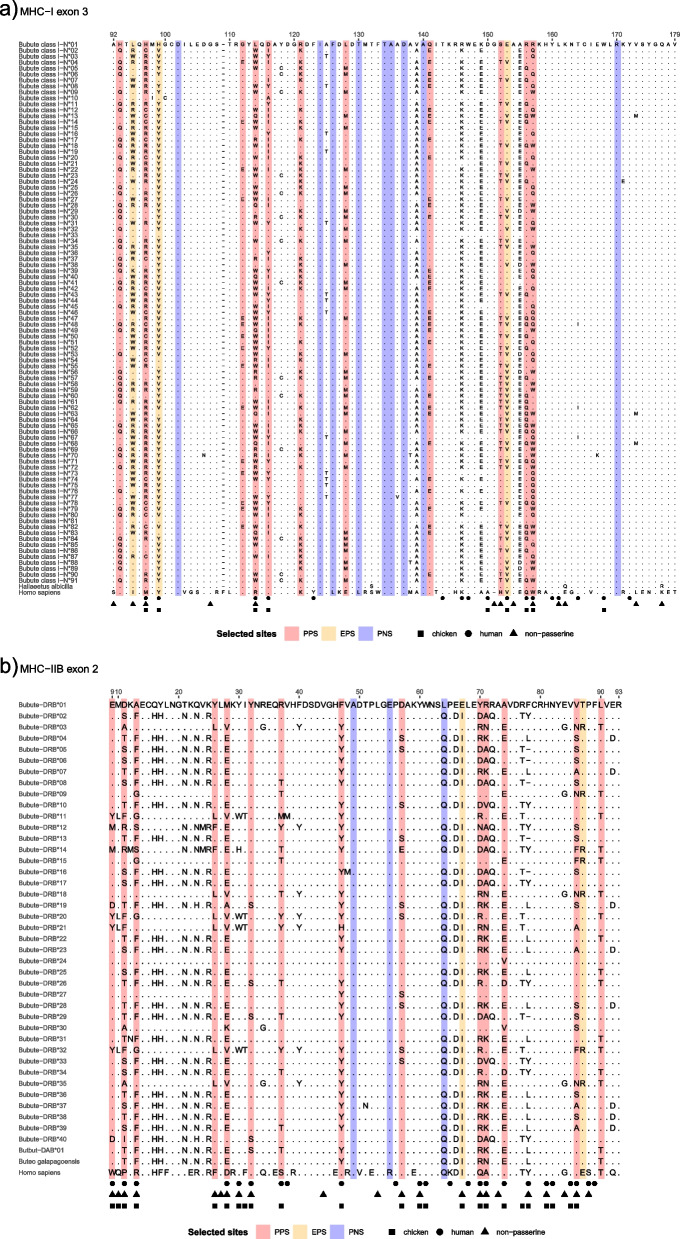
Alignments of amino acid sequences for MHC-I exon 3 and MHC-IIB exon 2 of common buzzards (*Buteo buteo*). **a**) Alignment of *Buteo buteo* MHC-I exon 3 putative alleles include sequences from human (accession # L06425) and white-tailed eagle (*Haliaeetus albicilla,* accession # MK186004), with numbering of residues 92–179 based on HLA-A2 alignment. **b**) Alignment of *Buteo buteo* MHC-IIB exon 2 putative alleles include sequences from human (accession # NP_002115) and Galapagos hawk (*Buteo galapagoensis*, accession # EU876825), with numbering of residues 9–93 based on HLA-DRB1 alignment. Dots indicate amino acids identical to the top sequence of MHC-I exon 3 and MHC-IIB exon 2, respectively. Circles represent human peptide binding residues, triangles represent inferred non-passerine peptide binding residues, and squares represent chicken peptide binding residues. PPS: pervasive positive selection, EPS: episodic positive selection, PNS: pervasive negative selection

Overall, signatures of selection were stronger in common buzzards for MHC-IIB exon 2 compared to MHC-I exon 3, in agreement with studies on the white-tailed eagle (*Haliaeetus albicilla*) and other non-passerines [34, 35]. Methods to detect pervasive and episodic selection (FUBAR and MEME, respectively) identified a higher number of sites under positive selection at MHC-IIB exon 2 compared to MHC-I exon 3, while MHC-I exon 3 had a higher number of sites under negative selection (Table 2, Fig. 2). Sites detected undergoing negative selection to maintain essential features of the protein generally showed no polymorphism across buzzard alleles. For example, Fig. 2a shows site 102 fixed for D (aspartic acid) across buzzard, white-tailed eagle, and human sequences. Sites detected undergoing positive selection generally showed changes in amino acids at multiple branches in the allele phylogeny. For example, Fig. 2a shows site 97 changing from the hydrophobic M (methionine) of the presumably ancestral lineage shared by humans and white-tailed eagle to R (arginine, basic), C (cysteine, polar neutral), and H (histidine, basic). This site is also identified as a PBR for humans and chickens, and hence amino acids changes here are likely adaptive.

**Table 2:** Signatures of selection at MHC-I and MHC-IIB exon 2 of common buzzards

			**Number of sites under selection**		**dN/dS and *P* for Z-test of positive selection (dN > dS)**									
**MHC class**	**Exon**	**n**_**seq**_** (n**_**AAsites**_**)**	**Pos**	**Neg**	**All sites**		**PSS**		**Non-passerine PBR**		**Human PBR**		**Chicken PBR**	
					**dN/dS**	***P***	**dN/dS**	***P***	**dN/dS**	***P***	**dN/dS**	***P***	**dN/dS**	***P***
MHC-I	2	10(87)	4	6	0.713	1.000	**3.336**	**0.004**			**3.293**	**0.007**	**2.579**	**0.013**
MHC-I	3	91(87)	14	9	1.054	0.429	**2.893**	**0.007**	1.137	0.394	1.708	0.181	1.733	0.140
MHC-IIB	2	41(85)	16	3	1.337	0.171	**11.472**	**1E-09**	**9.353**	**2E-04**	**7.457**	**0.001**	**5.152**	**2E-04**
MHC-IIB (no recomb.)	2	36(85)	15	3	1.366	0.162	**5.957**	**3E-10**	**6.622**	**2E-04**	**5.143**	**2E-05**	**5.058**	**5E-06**

Strength of selection was measured with the number of sites under pervasive and episodic positive selection (as inferred with FUBAR and MEME algorithms, respectively) and with the ratio of nonsynonymous substitutions per nonsynonymous site to synonymous substitutions per synonymous site, averaging over all sequence pairs (dN/dS) and Z-test of positive selection (dN > dS) at: (i) all sites; (ii) positively selected sites (PSS); (iii) putative peptide-binding residues (PBR) of non-passerines, as inferred from the global analysis of selection at the avian MHC [34]; and from the crystallographic structure of MHC-I and II molecules for (iv) human peptide-binding residues [19, 20] and (v) chicken peptide-binding residues [23, 59]. Estimates were inferred for all MHC-I and IIB sequences, and for MHC-IIB exon 2 alleles with non-recombinant segments (no recomb.). n_seq_: number of sequences, n_AAsites_: number of amino acid sites. Significant (*p* < 0.05) ratios of dN/dS are in bold

Measuring the strength of selection as the ratio of nonsynonymous and synonymous substitutions per site (dN/dS) also revealed MHC-IIB exon 2 to be under stronger diversifying (positive) selection than MHC-I exon 3 when considering positively selected sites (PSS), non-passerine PBRs, human PBRs, and chicken PBRs (Table 2). These patterns of number and signal of positively selected sites held when controlling for effects of recombination in MHC-IIB exon 2, which was expected because the proportion of sites matching for MHC-IIB exon 2 alignments with and without recombinant segments was high (proportion matching = 0.92).

When we looked at the first half of the peptide binding groove for MHC-I (exon 2), we identified 4 sites under positive selection and 6 sites under negative selection. Selection analysis revealed MHC-I exon 2 to be under significant, though lower diversifying selection (dN/dS) than MHC-IIB exon 2 at PSS, human PBRs, and chicken PBRs (Table 2, Additional file Figure S6). We did not extend the selection analyses to MHC-IIA exon 2 because three sequences would not provide statistical power to detect selection at single sites [60].

When we compared buzzard positively selected sites (PSS) with published peptide binding residues (PBRs), 7 out of 17 human PBRs at MHC-I exon 3 matched the 14 positively selected sites (PSS) in common buzzards (χ2 = 9.846, *P* = 0.005), 5 out of 13 non-passerine PBRs matched buzzard PSS (χ2 = 5.664, *P* = 0.032), and 7 out of 9 chicken PBRs matched buzzard PSS (χ2 = 28.289, *P* = 0.0001, Fig. 2a). At MHC-IIB exon 2, 11 of 24 human PBRs matched buzzard PSS (χ2 = 15.967, *P* = 0.0007), 11 out of 22 non-passerine PBRs matched buzzard PSS (χ2 = 18.881, *P* = 0.0002), and 14 out of 24 chicken PBRs matched buzzard PSS (χ2 = 34.165, *P* = 0.0001, Fig. 2b). These significant associations held when recombinant MHC-IIB segments were removed from the selection analyses (human PBR overlap: 8/24, χ2 = 5.662, *P* = 0.024; non-passerine PBR overlap: 10/22, χ2 = 15.793, *P* = 0.0003, chicken PBR overlap: 11/24, χ2 = 18.282, *P* = 0.0003). At MHC-I exon 2, all 4 positively selected sites in buzzards matched PBR identified in humans and chicken: 4 of 18 human PBRs matched buzzard PSS (χ2 = 16.520, *P* = 0.0009), and 4 out of 15 chicken PBRs matched buzzard PSS (χ2 = 20.662, *P* = 0.0007, Additional file Figure S6a). In summary, while both human and non-passerine PBR were good predictors of positively selected sites for MHC class I exon 2 and 3 and class IIB exon 2 in common buzzards, chicken PBRs were better predictors for both MHC classes overall.

### Phylogenetic diversity and relationships

**Fig. 3:**
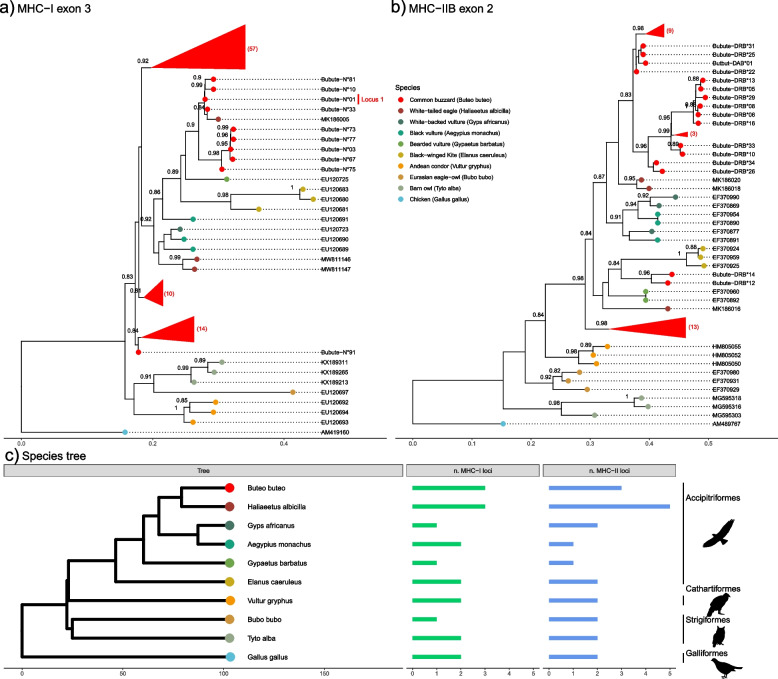
Phylogenetic relationships of common buzzard MHC-I exon 3 and MHC-IIB exon 2 alleles across closely related Afroaves species. Approximately-maximum-likelihood phylogenetic trees were created for **a**) 91 *Buteo buteo* alleles of MHC class I exon 3 and **b**) 41 *Buteo buteo* alleles of MHC class IIB exon 2. Scale bars indicate the number of substitutions per site. For clarity, triangles represent collapsed common buzzard clades with > 0.8 bootstrap support and red numbers in parentheses indicate number of sequences in each triangle. Full phylogenetic trees displaying all buzzard MHC-I exon 3 and MHC-IIB exon 2 sequences are provided as Additional file Figure S7 and S8, respectively. In a) the vertical red bar indicates the locus position that was fixed for the allele Bubute class I-N*01 exon 3. In **b**) the allele clade including alleles Bubute-DRB*13, 05, 29, 08, 06, and 16 was not collapsed to display the clustering of the alleles with a 3 bp deletion. **c**) The phylogenetic consensus tree of species included in the allele trees with the recorded maximum number of loci for MHC-I and MHC-II [data from 31, 62, 63, and the present study]. Species in the phylogenies are color-coded and include *Haliaeetus albicilla*, *Gyps africanus*, *Aegypius monachus*, *Gypaetus barbatus*, and *Elanus caeruleus* from the order Accipitriformes (osprey, kites, hawks, eagles), *Vultur gryphus* from the order Cathartiformes (New World vultures), *Bubo bubo* and *Tyto alba* from the order Strigiformes (owls and barn owls), and *Gallus gallus* as an outgroup from the order Galliformes (grouse, pheasants and partridges). Scale bar on the phylogenetic consensus tree indicates million years

**Fig. 4:**
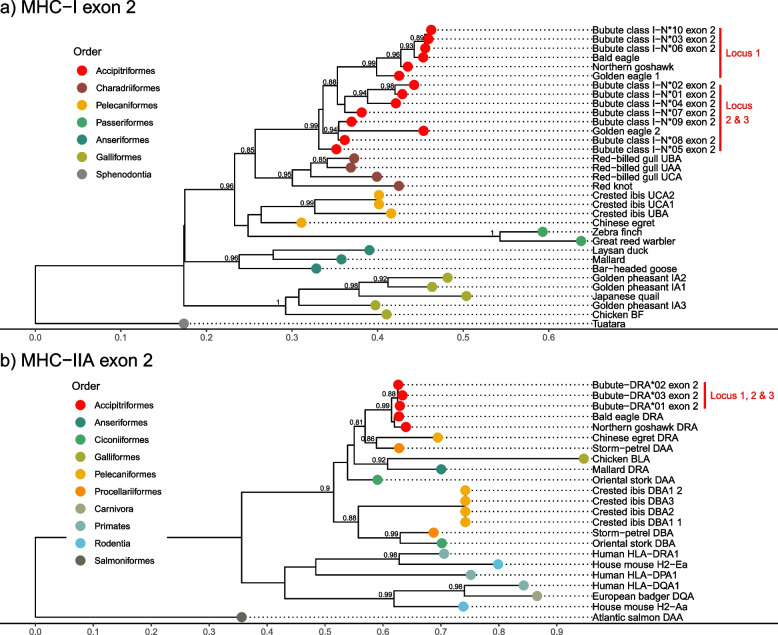
Phylogenetic relationships of MHC-I exon 2 and MHC-IIA exon 2 alleles among other avian orders. Approximately-maximum-likelihood phylogenetic trees were created for common buzzard (*Buteo buteo*) MHC alleles to infer the evolutionary history of gene duplication for class I and class II. Scale bars indicate the number of substitutions per site. Bootstrap values > 0.8 are shown. See Methods for accension numbers. **a**) MHC class I exon 2 alleles cluster by order, with common buzzard alleles clustering with Accipitriformes. Vertical red bars show the locus location of the alleles based on haplotype-aware long-read contigs. **b**) MHC class IIA exon 2 alleles tend to cluster by isotype (such as mammalian DRA and DQA and avian DBA) instead of order. Common buzzard alleles cluster with Accipitriformes, Pelecaniformes, and Procellariiformes (the DRA/DAA isotype) and locally by species with very short branch lengths. Together this suggests common buzzards share a DRA/DAA isotype with other orders, and recent duplications occurred in common buzzards

Phylogenetic inference of common buzzard (*Buteo buteo*) MHC-I exon 3 and MHC-IIB exon 2 shows that alleles from each class do not fall neatly into three clades (Fig. 3a, 3b), as would be expected for three locus copies that evolved independently. This lack of three clear clusters is repeated in phylogenetic relationships of MHC-I exon 2 and MHC-IIA exon 2 (Fig. 4a, 4b). This suggests that either gene duplication occurred too recently for alleles to differentiate or that that common buzzard alleles have not evolved independently and may be shared across loci or have undergone recombination (gene conversion). In support of gene conversion, we found evidence of recombination in MHC-I exon 2 and MHC-IIB exon 2. For MHC-IIB, it has been shown that sequence transfer between genes contributes to concerted evolution that has eroded the footprints of gene duplication for two ancient MHC-IIB lineages in avian taxa [61]. We also found evidence from genomic data that sequences are shared across loci for MHC-I exon 2 and MHC-IIA exon 2 (Additional file Table S3 and Table S4).

Phylogenetic relationships of exons do provide some evidence about the sequence and timing of gene duplications. Keeping in mind that both exon 2 and 3 make up the peptide-binding groove, MHC-I appears to have an ancient Locus 1 lineage shared within the order Accipitriformes with white-tailed eagles (*Haliaeetus albicilla*) (exon 3; Fig. 3a), and golden eagle (*Aquila chrysaetos*), bald eagle (*H. leucocephalus*) and northern goshawk (*Accipiter gentilis*) (exon 2; Fig. 4a). In contrast to Locus 1, Loci 2 and 3 do not align with monophyletic clades. For an example with MHC-I exon 2, Bubute class I-N*07 and N*08 are shared among Loci 2 and 3 and alleles Bubute class I-N*01, N*02, N*05, and N*09 are all found at Locus 2 but do not cluster together (Additional materials Table S3 and S4). However, Locus 2 contains Bubute class I-N*08 exon 2 and N*09 exon 2, which cluster with the golden eagle second locus at 0.94 support (Fig. 4a); thus, it is likely that a second gene copy was present in the common ancestor of common buzzards and golden eagles (separated by 33.4 myr) and the third gene copy arose recently in common buzzards. This idea of two shared MHC-I allelic lineages within the order Accipitriformes and a third gene copy arising in either common buzzards or in the common ancestor of close relatives is supported by Fig. 3c showing the estimated number of MHC-I loci in Accipitriformes to be 1–3. Phylogenetic patterns supporting the presence of two gene copies in Accipitriformes and then more recent duplications within common buzzards were consistent using alternative methods for phylogenetic reconstruction (Additional file Figure S9, S10, and S11).

In contrast to the more polymorphic exons, MHC-IIA exon 2 alleles tend to cluster by isotype (such as mammalian DRA and DQA and avian DBA) instead of order (Fig. 4b and Additional material Figure S11b). Common buzzard alleles of MHC-IIA exon 2 cluster at 0.99 support with closely related Accipitriformes, but also cluster distantly with Pelecaniformes and Procellariiformes at 0.81 support (the DRA/DAA isotype) and locally by species at 0.88 support with very short branch lengths. Together this suggests common buzzards share an ancient DRA/DAA lineage within Accipitriformes and possibly across orders, and recent duplications occurred in common buzzards.

While most MHC-I exon 2 and exon 3 and MHC-IIB exon 2 alleles clustered by species, phylogenetic analysis showed that some alleles were more closely related between species than within species. These phylogenetic patterns were consistent using alternative methods for phylogenetic reconstruction (Additional file Figure S9, S10, and S11). We highlight that common buzzard alleles only clustered within the order Accipitriformes but were widely distributed across the order. Common buzzard MHC alleles mostly clustered with the closest related species in our tree separated by 23.8 myr, the white-tailed eagle (*Haliaeetus albicilla*) and by 24.1 myr, the bald eagle (*H. leucocephalus*), though a MHC-I exon 3 sequence (Bubute class I-N*01) also clustered with the more distantly related bearded vulture *Gypaetus barbatus* (Fig. 3a and Figure S9) and MHC-IIB exon 2 sequences clustered with the distantly related white-backed vulture (*Gyps africanus*) and black vulture (*Aegypius monachus*) separated by 35.1 myr (Fig. 3b and Figure S10).

## Discussion

Counter to expectations from previous gathered evidence, we found relatively high copy number, allelic diversity, and sequence polymorphism in both MHC classes of a bird of prey. By genotyping over 100 individuals and employing genetic, genomic, and transcriptomic high-throughput sequencing methods, we were able to confirm that high population and within-individual allelic diversity is functional and identify three loci each for the MHC-I and MHC-II region in common buzzards. However, the large range of alleles recorded per individual as well as the lack of clear phylogenetic relationships among alleles make loci assignment challenging. Nevertheless, our study is one of the few on non-model species that considers both molecular domains‒coded by exon 2 and exon 3 for MHC class I genes and coded by exon 2 for MHC class IIA and B genes‒making up the peptide-binding groove for both MHC classes. In doing so, we find that purported higher sequence polymorphism and signatures of positive selection in MHC-II compared to MHC-I in non-passerines [34–36] only holds when viewing half of the peptide binding groove (MHC-I exon 3 for class I and MHC-IIB exon 2 for class II). The evidence of stronger selection on class II than class I in non-passerines disappears when we can see the entire picture. We next discuss our main findings and their implications.

### Allele characterization

Classical MHC molecules are defined by three essential properties: high polymorphism, high expression in many different cell types, and peptide presentation. It seems likely our sequences derive from classical MHC loci because they are polymorphic, well expressed from whole blood, and are functionally conserved at key peptide-binding residues, suggesting peptide presentation. However, population genotyping revealed that every single individual had the exon 3 allele Bubute class I-N*01. A few lines of evidence suggest that Bubute class I-N*01 exon 3 derives from a nonclassical locus. First, no functional polymorphism was observed across exon 2 and 3 sequences at Locus 1 (putative locus UAA, additional File Table S3). Second, sequence similarity and phylogenetic evidence suggest that the allele’s locus is conserved across closely related species. For example, Bubute class I-N*01 is most similar to the MHC-I exon 3 fragment of the white-tailed eagle (*Haliaeetus albicilla*), Haal-UA*01 allele (98.5% pairwise identity; ascension # MK186004). Mirroring our allele’s high frequency, Haal-UA*01 was found in over 90% of the 67 white-tailed eagle nestlings sampled [35]. Further, phylogenetic analyses show Locus 1 clustering with very short branches and high support with closely related species (Fig. 3a and 4a). Thus, the evidence suggests that Locus 1 (UAA) is a nearly monomorphic locus, conserved at the protein (functional) level and across species, and therefore likely a nonclassical locus**.**

### Genomic and expression data

Birds of prey and other non-passerines are thought to have relatively low MHC locus copy number and allelic diversity compared to passerines [31, 34, 52, 62, 63]. Previous studies have estimated between one and two MHC loci for most birds of prey, including MHC-IIB for common buzzards, based on PCR and cloning of sequences from few individuals [52, 62]. Therefore, our finding of one to at least three gene copies of both MHC classes in common buzzards from multiple forms of high-throughput sequencing data was unexpected, and strongly suggests CNV is present in this species. We also found tandem pairs of class IIA and IIB genes in our long-read data, consistent with tandem duplications seen in mammals [64] and in some non-model bird species [65–67]. By retrieving sequences from haplotype-aware contig long-read assemblies and blast-searching them against RNAseq transcripts, we were able to confirm that all three loci for each MHC class are expressed (Additional file Table S4). Interestingly, most individuals have 5 alleles at MHC-I exon 3 (Figure S2), and this fits with the genomic haplotype data showing that locus 1 is fixed for MHC Bubute class I N*01 exon 3 (assuming the other 2 loci are heterozygous; Additional file Table S3). Individuals exceeding 6 variants per genotype could either represent PCR/sequencing artefacts or copy number variation. Based on the abundance and expression of the alleles found in the genotypes of these high variant individuals, we believe that copy number variation is the more likely explanation.

Combining RNA sequencing with DNA amplicon sequencing revealed that alleles at high frequency in the population tended to be more prevalent in expression data. This effect is most likely due to sampling bias because our RNA sequencing study was not designed to test for expression in all MHC genotyped samples. However, it is interesting to observe that even alleles present at the lowest population frequency were often found in expression data. As DNA and RNA data were independently extracted and amplified, the dual presence of alleles in both datasets demonstrates that alleles found within single individuals can be verified as true, expressed alleles. Thus, rare alleles amplified from single individuals should not be immediately discarded as a way of more conservative genotyping, as this can underestimate true population allelic diversity.

Another insight generated from our expression data relates to MHC-IIB exon 2 alleles with a codon deletion that has been observed in related *Buteo* species (*Buteo galapagoensis* and *B. swainsoni* [63]), as well as in 21 non-passerine and passerine species distributed across phylogenetically distinct clades [34]. It was proposed that similar selection pressures may have favored the independent appearance of this codon deletion multiple times throughout avian MHC evolution [34]. Our study is the only one that tested for expression of alleles with this deletion, and we did not find evidence that any of the six alleles were expressed. This could indicate that either the codon deletion results in advantageous inactivation of the allele, or that expression of these six MHC-IIB exon 2 alleles depends on conditions that were not sampled in our RNAseq experiments. For example, MHC-II gene expression can be tissue-specific or depend on infection or inflammation [18].

### Polymorphism and signals of selection

We found greater sequence divergence and stronger positive (diversifying) selection measured as number of positively selected sites and dN/dS at MHC-IIB exon 2 than MHC-I exon 3. Stronger measures of balancing selection for MHC-IIB exon 2 than MHC-I exon 3 were also found in other non-passerine species [34–36] and one explanation has been that different modes of selection act upon MHC-I and MHC-II depending on passerine or non-passerine status [34, 68]. However, previous studies have compared only these two exons between the classes, essentially considering only half of the sequence encoding the peptide-binding groove. By including MHC-I exon 2 and MHC-IIA exon 2, the other molecular domains completing the peptide binding groove of MHC class I and II, respectively, we could see the whole picture. This revealed that both sequence polymorphism and measures of balancing selection were similar for MHC-I exon 2 and exon 3, shown previously in a comparative study using 20 avian families [37]. In fact, polymorphism was higher at MHC-I exon 2 than exon 3, on par with polymorphism at MHC-IIB exon 2 (Table 1). In contrast, MHC-IIA exon 2 was almost monomorphic, seen in other vertebrates including humans and non-human primates [39] and non-passerines like chicken [38] ducks [69], and storm petrel [65]. Essentially, this means that two polymorphic domains encode the peptide-binding groove for MHC class I, while one polymorphic domain and one monomorphic domain encode the peptide-binding groove for MHC class II. Table 3 demonstrates how false conclusions can arise from comparing single domains between MHC classes. For example, in humans, MHC class I exon 2 and 3 each show similar nucleotide diversity values and measures of diversifying selection (dN/dS) with MHC class II DRB1 exon 2. However, when both peptide binding domains are compared between MHC-I and II, then MHC-I is much more diverse than MHC-II and shows almost significantly stronger diversifying selection (*P* = 0.059). Indeed, comparing single exons in buzzards results in significantly stronger selection at MHC-IIB than MHC-I exon 3, but seemingly higher sequence diversity and stronger selection vanish when both domains are compared between the classes.

**Table 3:** Comparing sequence polymorphism and strength of selection between MHC classes using both peptide binding domains

Species	MHC region	n_alleles_	Pi	dN/dS	dN-dS	SE	Z-score	*P*
Human	HLA-I exon 2 (α1)	83	0.087	3.160	0.203	0.081	0.728	0.233
	HLA-I exon 3 (α2)	83	0.065	2.447	0.123	0.076	0.052	0.479
	*HLA-I exon 2 & 3 (α1α2)*	*83*	*0.076*	*2.809*	*0.161*	*0.054*	*1.565*	*0.059*
	HLA-II DRA exon 2 (α1)	1	0.000	NA	0.000	0.000		
	HLA-II DRB1 exon 2 (β1)	29	0.062	1.922	0.117	0.086		
	*HLA-II DR- A & B1 exon 2 (α1β1)*	*29*	*0.033*	*1.750*	*0.050*	*0.046*		
Common buzzard	MHC-I exon 2 (α1)	10	0.094	3.461	0.219	0.075	-1.147	0.126
	MHC-I exon 3 (α2)	8	0.065	2.635	0.121	0.059	**-2.042**	**0.021**
	*MHC-I exon 2 & 3 (α1α2)*	*10*	*0.079*	*3.089*	*0.166*	*0.042*	*-0.143*	*0.443*
	MHC-IIA exon 2 (α1)	3	0.008	NA	0.035	0.036		
	MHC-IIB exon 2 (β1)	8	0.105	9.156	0.367	0.105		
	*MHC-II A & B exon 2 (α1β1)*	*8*	*0.056*	*7.481*	*0.175*	*0.047*		

To demonstrate how a false conclusion can be reached when comparing only one set of peptide binding domains between MHC classes, we collected and analyzed well-curated alleles from humans and full alleles of common buzzards. Human class I HLA-A, B, C, and class II HLA-DRB1 alleles from the European population classified as "*common*" in the Common and Well-Documented allele catalog 3.0.0 [70] as well as HLA-DRA*01:01 from the monomorphic DRA locus were downloaded from IPD-IMGT/HLA Database version 3.51 [16] (Additional file Table S5). Common buzzard alleles with full peptide-binding grooves were retrieved from genomic data (GenBank accession #s: OL311287, OL311290, OL311292, OL311294, OL311304, OL311305, OP490259, OQ414190-OQ414202, OQ428163-OQ428174). All analyses for the strength of selection (dN and dS) were conducted in MEGA X [71] using human peptide binding residues from [72] and the Nei-Gojobori model [73] with 1000 bootstraps for variance estimation. The Z-score was calculated with the formula (dN-dS_MHC-I_—dN-dS_MHC-II_)/$\surd$(SE^2^_MHC-I_ + SE^2^_MHC-II_). We compared MHC-I exon 2 and 3 separately with MHC-IIB exon 2, and then combined against concatenated MHC-IIA & B exon 2 sequences. We tested if MHC-I had stronger selection than MHC-II in humans and if MHC-IIB had stronger selection than MHC-I in common buzzards. One-sided *p*-values were generated from the Z-scores. *P*-values show how comparing single exons between the classes does not represent comparisons across the full binding domains (*italicized for emphasis*; MHC-I: α1α2, MHC-II: α1β1). n_alleles_: number of alleles, Pi: nucleotide diversity, SE: standard error of the mean. Significant *p*-values are highlighted in bold

In summary, we found that looking at the complete peptide binding groove of each class revealed no difference in polymorphism or the strength of balancing selection between MHC-I and MHC-II in a non-passerine. We could not find any study on MHC-IIA exon 2 in passerines, so we do not know if monomorphism is the pattern across birds. But if MHC-IIA monomorphism holds more generally, it would argue against differential selection at MHC classes between passerines and non-passerines. Allelic diversity was still much higher at MHC-I (10 alleles at exon 2, 91 alleles at exon 3) than MHC-II (3 alleles at IIA, 41 alleles at IIB) which could indicate that different modes of pathogen-mediated selection are acting on the two classes requiring many ‘specialist’ alleles for class I and fewer ‘generalist’ alleles for class II [23, 24, 74].

Another key finding of our study was that chicken peptide binding residues (PBR) determined by crystallographic structure analysis of MHC-I and II molecules [23, 59] significantly improved matching rates of buzzard positively selected sites compared to using human PBR [19, 20] or PBR inferred from a global analysis of 296 non-passerine species [34]. Thus, for avian MHC studies we support the use of chicken PBR identified using mechanistic analysis of MHC molecular structure.

### Phylogenetic relationships

To investigate the phylogenetic relationships among MHC of common buzzard and closely related Afroaves species, we created MHC gene sequence trees and compared these with species trees. Although long-read sequencing data displayed three loci each for common buzzard MHC-I and MHC-II, our phylogenetic inference did not show three distinct clades of alleles for each class. This suggests that either common buzzard MHC genes duplicated too recently to show orthologous clustering of alleles, or that alleles did not evolve independently after gene duplication. For example, alleles could be shared across loci or have undergone inter-locus gene conversion leading to homogenization [61], for which there is evidence of recombination for MHC-IIB exon 2 and MHC-I exon 2. Alternatively, if selection for broader parasite surveillance promotes divergence of alleles within loci (‘divergent allele advantage’) [22, 75], then alleles will not necessarily cluster phylogenetically. In fact, common buzzard alleles do not even cluster by species, a phenomenon known as trans-species polymorphism [27]. This pattern of some alleles being more phylogenetically related between than within species is common to MHC genes―and immune genes in general―that are subject to parasite-mediated balancing selection preserving allelic diversity over long time periods [28]. However, convergent evolution driven by similar selective (e.g., parasite) pressures could also lead to shared polymorphisms between independently evolved lineages [76, 77].

We were able to identify one MHC-I locus (UAA Locus 1) that is shared among the Accipitriformes order and is at least 33.4 million years old based on divergence from the golden eagle (*Aquila chrysaetos*). There is phylogenetic evidence that a second MHC-I locus is also shared within the order Accipitriformes, and that a third locus arose via gene duplication in either common buzzards or in the common ancestor of close relatives. In contrast to the relatively young ages of MHC-I allele lineages, the MHC-IIA DR-like lineage shared by common buzzards, chickens, and ducks is ancient, predicted to be at least 300 million years old and preserved by purifying selection to conserve functionality [38].

### Limitations

Our study used one of the most stringent genotyping protocols in contemporary studies of MHC characterization. Namely, by including a high percentage of technical replicates, confirming expression of many alleles using MHC-genotyped and additional samples, and using genomic data to verify the number of loci at MHC-I and MHC-II. However, with all these quality controls, we still had difficulty assigning all sequences to loci using haplotype-aware HiFi contigs from a few individuals and using maximum-likelihood (ML) haplotyping methods on genotype data. This inability to assign all alleles to loci prevents us from testing for inter-locus gene conversion and from determining if indeed alleles are shared across loci or if copy number variation (CNV) is present within common buzzards. Similar genotyping studies should either use thousands of samples for ML haplotyping [51] or combine long-read technology [78] and allele segregation patterns in families [79, 80] to identify if and to what extent CNV occurs in populations.

## Conclusions

Overall, using genetic, genomic, and transcriptomic high-throughput sequencing data we have shown that common buzzards, an Accipitriform bird of prey, has at least triplicated MHC class I and II loci. Furthermore, the documented expression of alleles at each locus confirms their functionality. There is still the question of the number of genes per animal and the expression of all alleles which should be followed up to provide a definitive account of gene copy number and their polymorphic content in this species. Sequence polymorphism and selection analyses appeared to support findings from comparative studies showing MHC-II has greater sequence divergence as well as stronger signatures of diversifying selection than MHC-I. However, upon further investigation, this turned out to be false. Both classes have similar levels of sequence polymorphism and diversifying selection once both molecular domains of the peptide-binding groove are considered. This result may hold more generally once more data on understudied MHC exons becomes available. Finally, phylogenetic analyses reveal trans-species polymorphism of common buzzard alleles, consistent with selection preserving adaptive alleles across taxa.

## Methods

### Population sampling

We sampled 130 common buzzard (*Buteo buteo*) chicks from 2004–2018 in a 300 km^2^ study area (8°25'E and 52°6'N) in Eastern Westphalia, Germany. Full trapping methods are described in [81]. Chicks were ringed, recorded for biometric measures, and a 0.5 ml blood sample was taken from the brachial vein with a syringe or needles and capillaries. Blood for DNA and RNA extraction was transferred into 1.5 ml screw-cap tubes filled with 1.0 ml ethanol, phosphate-buffered saline–EDTA buffer or RNAlater and stored at − 20 °C or -80 °C, respectively. Sampling methods were approved by the Animal Ethics Committee at Bielefeld University and conducted with permission from the local authority Kreis Gütersloh, permit number: 4.5.2–723-Bussard and from the federal state authority 84–02.04.2014.A091, 84–02-04.2017.A147 in accordance with German federal and state laws.

### DNA and RNA extraction

DNA was extracted using a standard chloroform protocol [81] and samples were normalized to 20 ug/uL for PCR. Total RNA was extracted from (whole blood) using innuPrep Blood RNA kit according to the manufacturer’s recommendations (Analytik Jena).

### PCR amplification and cloning

The MHC class I α2 domain (303 bp with primers included) encoded by exon 3 was amplified using primers MHCI-int2F (5'-CATTTCCCTGGTYGTGTTTCAGG-3'; [62]) designed for birds of prey and MHCI-ex3R (5’- CTCACCTTTCCTCTCCAG-3’; [82]) designed for Falconiformes.

A 297 bp fragment (primers included) of the MHC class II β1 domain encoded by exon 2 was amplified using primers Acc2FC (5'-GCACAAACAGGGTTYTTCC-3'; [52]) designed for diurnal raptors and ButeoR (5’-TTCTGGCACRCACTCACCTC-3’; [63]) designed for the genus *Buteo*.

PCR amplification was carried out in 20 μl reactions using 1X S buffer, 0.8 mM dNTPs, 0.25 μM of each primer, 1U PeqGold Taq DNA polymerase (VWR Peqlab), and 10–50 ng of genomic DNA. Reaction conditions were as follows for MHC-I exon 3 and MHC-IIB exon 2: (MHC-I) 96 °C for 2 min, then 30 cycles of 94 °C for 30 s, 60 °C for 30 s, and 72 °C for 1 min, and a final extension of 72 °C for 10 min; (MHC-II) 94 °C for 4 min, then 35 cycles of 94 °C for 40 s, 58 °C for 40 s, and 72 °C for 1 min, and a final extension of 72 °C for 5 min. We ran 5 μl of PCR product per reaction on 2% agarose gels to confirm amplification. We used ExoSAP-IT cleanup to purify the PCR products and then cloned them using the TOPO® TA Cloning® Kit for Sequencing (Invitrogen). Positive clones were Sanger sequenced using BigDye chemistry (Applied Biosystems). Sequences were confirmed as putative alleles if present in two or more cloned sequences and if they were highly similar (< 1e^−5^) via Megablast to MHC sequences from birds of prey. PCR products of clones representing these putative alleles were used as positive controls for high-throughput sequencing.

### Library prep and high-throughput sequencing

After cloning confirmed our primer pairs amplify *Buteo buteo* MHC sequences, we performed PCR tagging for 130 individuals for MHC-I exon 3 and MHC-IIB exon 2, 5 clones, and 3 control wells (no DNA), with technical replicates for 95 individuals. 10 bp tags with an edit distance of 7 [83] were combined with 8F and 12R primers to uniquely label individuals so that they could be pooled within a single library (i.e., one Illumina adapter was used per plate). 6–8 bp of ‘junk’ DNA was added to the 5’ direction of the tagged primers to provide complexity for Illumina sequencing. Altogether, primers consisted of 5’-junk‒tag‒primer-3’.

We simultaneously purified and normalized PCR amplicon concentrations to ~ 4.4 ng/μl (Norgen NGS 96-Well Kit), pooled, and quantified pools using Qubit dsDNA BR Assay Kit for 2.0 Fluorometer (Life Technologies). We used between 24 – 74 ng (in 200 μl) of pools for Illumina TruSeq NanoDNA LS library construction. After purifying libraries to remove adapter products and validating libraries (Bioanalyzer Agilent DNA 2100 kit) we confirmed we had between 1.7 – 4.1 nM library concentrations for Illumina MiSeq sequencing. Sequencing was performed in a single run using the MiSeq® Reagent Kit v2 (500 cycle) at the Max Planck Institute for Evolutionary Biology, which included 30 libraries for another experiment and six for common buzzards. Some adapters were used multiple times, but always in combination with different primers for downstream demultiplexing. To confirm the reliability of our genotyping pipeline, we also Illumina sequenced five unique clones of MHC-I exon 3 with individual tags, and all five clones had a single allele per genotype. ‘Negative controls’ were not assigned any alleles and confirm that contamination was not an issue during sequencing.

### Processing data and MHC allele validation

Raw fastq files were pre-processed, demultiplexed, clustered, and filtered using the online AmpliSAT pipeline (http://evobiolab.biol.amu.edu.pl/amplisat/index.php). AmpliMERGE was used to merge paired-end reads from each library and AmpliCLEAN was used to filter reads not belonging to any amplicon and to remove low quality reads (below a minimum Phred quality score of 30). After pre-processing, demultiplexing and quality filtering, Library 1 had 920,315 reads, of which 153,579 reads were kept; Library 2 had 247,777 reads, of which 54,867 reads were kept; Library 3 had 243,009 reads, of which 55,852 reads were kept. AmpliCHECK and AmpliCOMPARE were then used to find the parameters for AmpliSAS clustering that would provide the highest genotyping reliability using technical replicates. AmpliSAS was used for de-multiplexing, clustering, and filtering of sequence variants [84]. AmpliSAS was run for MHC-IIB exon 2 using fasta sequences of alleles previously recorded for *Buteo buteo* (GenBank ascension #s EF370899, EF370900, EF370956, KJ162430). The minimum read number per amplicon was set to 100 and the maximum allele number was set to six because long-read genomic data identified three loci for both MHC-I exon 3 and MHC-IIB exon 2 (detailed below). All AmpliSAS parameter settings can be found in Additional file Table S1.

After genotyping, putative alleles were validated through a series of steps. First, we used Megablast on putative alleles to examine similarity to known MHC alleles from other species. Second, we translated putative alleles to check for stop codons and to identify regions of conserved structural importance for classical MHC loci [48, 85]. Third, we examined the mean percent reproducibility among technical replicates, calculated as [(Number of shared alleles*2)/sum of alleles in replicates)]. Finally, we confirmed putative allele expression with RNA-seq experiments.

### MHC allele expression

Genomic RNA was sequenced for another study from 81 individuals, 37 of whom also had MHC genotype data. Details of RNA sequencing methods can be found in Rinaud T, Ottensmann M, Krueger O, Winternitz J, Chakarov N, (*unpublished data*). Briefly, library preparations from whole blood RNA extractions were shipped on dry ice and sequenced on Illumina NovaSeq™ 6000 at the Beijing Genomic Institute (BGI), China. They performed short paired-end sequencing and standard quality control. Raw reads were assembled de novo using the Trinity suite standard parameters (https://github.com/trinityrnaseq/trinityrnaseq). Buzzard-specific transcripts were mapped from whole assemblies to chicken (GCA_016699485.1) and golden eagle (GCA_900496995.4) genomes and to our own buzzard draft genomes (for additional buzzard specific transcripts, *unpublished*). MHC-I and MHC-II sequences were compared to buzzard transcripts using Megablast with stringent cut-offs of 100% percentage identity and e-value of 1e-40.

### Copy number determined using genomic long-read data

gDNA was collected from four individuals in the same 300 km^2^ study area (8°25'E and 52°6'N) in Eastern Westphalia, Germany, during 2020 for genomic inference. PacBio HiFi sequencing produced 2.3 M sequencing reads and 37.6 Gb bases of sequence which corresponds to approximately 27-fold coverage of the *B. buteo* genome. The average read length was N50/N90: 16,856/12558 with an average RQ of 72. Mean coverage of HiFi reads per individual was 14. To organize the genome by chromosome, Hi-C scaffolding was conducted for three individuals with Phase Genomics and Illumina sequencing of Hi-C reads with average coverage of 90 per individual. All sequencing was done at the West German Genomics Center Düsseldorf/Cologne. HiFi assembly was done with Flye [86], Hi-C with juicebox and DNA3d of the Aiden Lab (https://github.com/aidenlab) using the default settings. All sequencing data presented are available on the figshare repository (DOI: 10.6084/m9.figshare.16885255). To identify the MHC region in the buzzard genome, known avian MHC sequences (GenBank accession #s: AB119993, AB872442, CHKMHBFVB, CHKMHCBCHA, EF370956, EU442606, HM008713, HM008714, HM008715, KC282841, KC282842, KC282843, KC282844, KP182409, KY511591, KY511592) were BLAST matched at equal/greater than 70% similarity onto buzzard hybrid PacBio HiFi-Hi-C scaffold assemblies. Both MHC-I and MHC-II regions mapped to scaffold_33/chromosome 29 (1,742,570 bp) using Minimap2 [87] with the preset PacBio settings. This allowed us to confirm that MHC class I exon 3 and class II DRB exon 2 are present in at least three locations in *Buteo buteo* ch29 (Additional file Figure S5). One individual (HiFi ID 326; contig_1060) appears to show four DRB exon 2 copies (and four DRA exon 2 copies), providing evidence that gene copy number variation is present in common buzzard MHC-II. To reconstruct MHC haplotypes from the HiFi data, haplotype-aware contigs of the MHC region were assembled with HiFi reads using Phasebook [88] with the parameters -t 8 -p hifi -g small -x -min_cov 2 -min_cluster 2 -min_allele_cov 2. Haplotypes of MHC class I exon 3, exon 2, and class IIB and IIA exon 2 are presented in Additional File Table S3.

We had originally planned to only focus on MHC-I exon 3 and MHC-IIB exon 2, two well-studied exons encoding their half of the peptide-binding groove. Additionally, these exons are believed by some researchers to be responsible for the majority of MHC functional polymorphism and the main targets of pathogen-mediated selection [37–39]. However, with haplotype-aware sequence data aligned to both regions of common buzzard MHC, we were able to retrieve the other halves of the peptide binding groove for MHC-I (exon 2) and MHC-II (MHC-IIA exon 2) to have a clearer picture of the peptide binding region for each class. The MHC class I α1 domain (264 bp) encoded by exon 2 was retrieved by Blast-searching avian MHC-I exon 2 against our haplotype-aware contigs aligned to buzzard chr 29 (accession #s: AB119993, M31012, HM008713, HM008714, HM008715, KP182409, KY511591, KY511592). Similarly, the MHC class IIA gene α1 domain (258 bp) encoded by exon 2 was retrieved from the same set of contigs by Blast-searching avian MHC-IIA exon 2 (accension #s: NM_001310349, NM_001245061, MN061408, MN061399, MN061393, MK981897, MK981896, MK829176, KF041454, HQ203731). Haplotypes of MHC class I exon 2 and class IIA exon 2 are presented in Additional file Table S3.

### Sequence polymorphism

Sequence polymorphism was assessed as the number of polymorphic sites, total number of mutations, average number of nucleotide differences, and nucleotide diversity using DnaSP v.6.12.03 [89]. The amino acid distance was measured as the amino acid p-distance with uniform rates in MEGA X [71]. Pairwise identity for MHC-I and II alleles was calculated using Geneious Prime 2021.2.2 (https://www.geneious.com).

### Recombination

Recombination was tested for using GARD [57] on the Datamonkey server (http://datamonkey.org/) and using RPD4 v.4.101 software [58], which implements several different algorithms developed to detect recombinant sequences. The following approaches were used to assess recombination in our data: RDP [90], GENECONV [91], BootScan [92], Maxchi [93], Chimaera [56], SiScan [94], and 3Seq [95]. MHC-I exon 3 and MHC-IIB exon 2 nucleotide sequence alignments were first screened using an automated exploratory search for recombination signals using the default settings, a statistical significance threshold of *P* = 0.05, and Bonferroni correction for multiple comparisons. A recombination event was recognized when supported by two or more algorithms. Next, we proceeded with manual examination following the guidelines of [96]. Briefly, we sequentially examined all detected recombination events for the following: the characteristics of a particular recombination event (i.e., its breakpoint positions and the identity of the recombinants) could be verified, the recombination event was detected in more than one sequence, the average p-value across recombinant sequences for the method detecting recombination was less than 0.05, and there was no warning that the apparent recombination signal could have been caused by an evolutionary process other than recombination. If all these criteria were met then the recombination event was accepted, if not, the event was rejected. Following acceptance of an event, the sequence alignment was iteratively rescreened, and the process repeated until all recombination events were checked. When recombination was verified, RDP4 was used to export sequence alignments with recombinant fragments of sequences removed for selection inference.

### Inference of selection

For MHC-I exon 2 and 3 and MHC-IIB exon 2, maximum likelihood fits for nucleotide substitution models was implemented in MEGA X [71]. BIC-based model selection identified K2 + G + I (I = 0.59, G = 0.34, *R* = 1.69) as the best model for MHC-I exon 3, T92 + G (G = 0.24, *R* = 1.55) for MHC-I exon 2, and JC + G + I (I = 0.54, G = 0.69, *R* = 0.50) as the best model for MHC-IIB exon 2. Selection was inferred using the popular method of comparing rates of nonsynonymous (dN) to synonymous substitutions (dS). The basic interpretation is that (dN > dS) implies positive (diversifying) selection driving changes in amino acids, (dN < dS) implies negative (purifying) selection against changes in amino acid, and (dN = dS) implies neutral evolution.

Codon sites under natural selection were detected by inferring relative rates of dN and dS on a per-site basis using two complimentary methods: FUBAR and MEME. FUBAR (Fast, Unconstrained Bayesian AppRoximation) uses a Bayesian approach to infer rates and assumes constant pressure across sites to detect evidence of pervasive selection [97]. It is possible that individual sites may experience different levels of positive and negative selection (i.e., episodic selection), which methods that detect only pervasive selection will miss. MEME (Mixed Effects Model of Evolution) employs a mixed-effects maximum likelihood approach to detect individual sites that have been subject to episodic and pervasive positive selection [98]. Therefore, we recorded positively selected sites (PSS) as those identified by either FUBAR (pervasively) or MEME (pervasively and episodically). Negatively selected sites were identified by FUBAR only. FUBAR posterior probabilities > 0.9 are strongly suggestive of natural selection [97] and this was set as our significance threshold. Our significance threshold for MEME was set to *p* < 0.05. Both selection inference methods were conducted on the Datamonkey server (http://datamonkey.org/).

The strength of selection was measured as mean dN/dS averaging over all sequence pairs and the significance was tested using codon-based Z-tests of positive selection (probability dN > dS) using 1000 bootstraps for variance estimation. For MHC-I exon 2 and 3, analyses were conducted using the Pamilo-Bianchi-Li (Kimura 2-parameter) method [99] with rates among sites Gamma distributed (shape parameter = 0.34) with invariant sites (G + I). The Pamilo-Bianchi-Li (Kimura 2-parameter) gamma model was chosen because it corrects for multiple substitutions and considers unequal transition and transversion rates (R) as well as differences in substitution rates among sites. For MHC-IIB exon 2, analyses were conducted using the Nei-Gojobori model with Jukes-Cantor correction for multiple substitutions [73] with rates among sites Gamma distributed (shape parameter = 0.69) with invariant sites (G + I) and pairwise deletion of gaps. All analyses for the strength and significance of selection (dN/dS) were conducted in MEGA X [71].

Positions of positively selected codon sites (inferred with both FUBAR and MEME analysis of sequence alignments) were compared to the classification of peptide binding residues (PBRs) identified in humans, non-passerines, and chickens. Human PBRs were based on the crystallographic structure of human MHC molecules [19, 20]; non-passerine PBRs were based on a global analysis of codon-specific signatures of positive selection of non-passerines [34]; chicken PBRs were based on the crystallographic structure of chicken MHC molecules [23, 59]. We tested if positively selected *Buteo buteo* residues were significantly associated with human, non-passerine, or chicken PBRs using permutation (randomization) tests for independence of two variables, with chi-square as the test statistic. We used the function perm.ind.test from the wPerm v1.0.1 R package [100] with 10,000 replications to test if the observed proportion of matching residues differed significantly (alpha = 0.05) from the distribution of expected random proportions. Tests were conducted separately for MHC-I exon 3 and MHC-IIB exon 2. Allele alignment figures were created using ggplot2 v3.3.5 [101].

### Phylogenetic relationships

To investigate the phylogenetic relationships among MHC of common buzzard and closely related Afroaves species [102], we searched for species from the Orders Accipitriformes (osprey, kites, hawks, eagles), Cathartiformes (New World vultures), and Strigiformes (owls and barn owls), that had both MHC class I exon 3 and class II exon 2 data available on GenBank (search performed on Aug. 18, 2021). We identified five species from Accipitriformes (*Aegypius monachus*, *Elanus caeruleus*, *Gypaetus barbatus*, *Gyps africanus*, *Haliaeetus albicilla*), one from Cathartiformes (*Vultur gryphus*), and two from Strigiformes (*Bubo bubo*, *Tyto alba*) with both classes of MHC data. Chicken (*Gallus gallus*) sequences were included as an outgroup. Multiple alignment was performed for each MHC class separately with Clustal Omega using Geneious Prime 2021.2.2 (https://www.geneious.com). After removing pseudogenes and trimming alignments to 262 bp and 258 bp for MHC-I exon 3 and MHC-IIB exon 2, respectively, we randomly drew three sequences per species from those that had more than three sequences available.

To create phylogenetic trees for MHC-I exon 2 we retrieved sequences from the family Accipitridae, order Accipitriformes, from the Northern goshawk *Accipiter gentilis* (XR_007504809), Golden eagle *Aquila chrysaetos* (XM_030006826, XM_030006833) and Bald eagle *Haliaeetus leucocephalus* (XM_010571240). As there were no other MHC-I exon 2 sequences available from the Orders Accipitriformes, Cathartiformes, or Strigiformes, we retrieved Genbank archived sequences from Charadriiformes: Red-billed gull *Chroicocephalus scopulinus* (HM008713, HM008714, HM008715); Red knot *Calidris canutus* (KC205115); Anseriformes: Laysan duck *Anas laysanensis* (KF612477); Mallard *Anas platyrhynchos* (AB119993); Bar-headed goose *Anser indicus* (FJ606105); Galliformes: Japanese quail *Coturnix japonica* (AB005527); Golden pheasant *Chrysolophus pictus* (KM005646, KM005648, KJ997735); Chicken *Gallus gallus* (KF032302); Passeriformes: Zebra finch *Taeniopygia guttata* (XM_002186531); Great reed warbler *Acrocephalus arundinaceus* (AJ005507); Pelecaniformes: Chinese egret *Egretta eulophotes* (KY511591); Crested ibis *Nipponia nippon* (KP182409); and we used Tuatara *Sphenodon punctatus* (DQ145788) as an outgroup. Sequences were aligned to preserve coding regions (CDS) of exon 2 using MAFFT v7.490 [103, 104] with Geneious Prime 2021.2.2 (https://www.geneious.com) and trimmed to the length of our buzzard sequences (MHC-I exon 2: 280 bp with gaps).

For MHC-IIA exon 2, we retrieved sequences from the family Accipitridae, Order Accipitriformes, from the Northern goshawk (*A. gentilis*; XM_049793033) and Bald eagle (*H. leucocephalus*; XM_010569905). We combined these with Genbank archived sequences from Ciconiiformes: Oriental stork *Ciconia boyciana* (LC180358); Anseriformes: Mallard *A. platyrhynchos* (HM070250); Galliformes: Chicken *G. gallus* (HQ203731); Pelecaniformes: Chinese egret *E. eulophotes* (KF041454); Crested ibis *N. nippon* (KP182408, MK829176); Procellariiformes: Storm-petrel *Oceanodroma leucorhoa* (MK981896; MK981897); and mammals: European badger *Meles meles* (HQ908097); House mouse *Mus musculus* (NM_010378, BC106107); Human *Homo sapiens* (NM_033554, NM_002122, NM_019111); with Atlantic salmon *Salmo salar* (L77086.1) as the outgroup. Sequences were aligned to preserve CDS using MUSCLE 3.8.425 [105] with Geneious Prime 2021.2.2 (https://www.geneious.com) and trimmed to the length of our buzzard sequences (MHC-IIA exon 2: 269 bp with gaps).

The phylogenetic trees for MHC alleles were created using FastTree v2.1.11 [106]. FastTree infers approximately-maximum-likelihood phylogenetic trees from sequence alignments, using the Jukes-Cantor model of nucleotide evolution with 20 rate categories of sites. To estimate the reliability of nodes, local support values were computed using 1000 resamples with the Shimodaira-Hasegawa test [107].

To compare the inferred MHC gene trees with the species tree, 1000 trees based on the Ericson All Species backbone tree [108] were downloaded from the Bird Tree website [109], http://birdtree.org/; accessed Aug. 18, 2021] for the ten species in this study. Consensus topology and average branch lengths were computed with the *consensus.edges* function from the R package phytools v0.7–80 [110] using 50% majority rule consensus tree. Numbers of recorded/inferred loci for MHC-I exon 3 and MHC-IIB exon 2 were compiled from [31, 35, 62, 111], and the present study. Phylogenetic tree figures were created using the R package ggtree v3.0.2 [112]. Analyses and figures produced in R used version 4.2.1 [113].

## Supplementary Information

**Additional file 1: Table S1.** AmpliSAS parameter settings. **Table S2.** Recombination signal at MHC class I exon 3 and MHC class II exon 2 of common buzzards. **Table S3.** Confirmed MHC haplotypes based on long-read sequencing in common buzzards. **Table S4.** RNA transcript data confirms all three MHC loci are expressed. **Table S5.** HLA sequences used for Table 3. **Figure S1.** Common buzzard MHC class I exon 3 alleles translation with evolutionarily conserved sites. **Figure S2.** Common buzzard MHC class I exon 3 allele count. **Figure S3.** Common buzzard MHC class IIB exon 2 alleles translation with evolutionarily conserved sites. **Figure S4.** Common buzzard MHC class IIB exon 2 allele count. **Figure S5.** Common buzzard MHC loci copy number inferred from long-read sequencing. **Figure S6.** Alignments of amino acid sequences for MHC class I exon 2 and MHC class IIA exon 2 of common buzzards. **Figure S7.** Phylogenetic relationships of common buzzard MHC-I exon 3 alleles across closely related Afroaves species. **Figure S8.** Phylogenetic relationships of common buzzard MHC-IIB exon 2 alleles across closely related Afroaves species. **Figure S9.** Phylogenetic relationships of common buzzard MHC-I exon 3 alleles across closely related Afroaves species from ML-based phylogenies. **Figure S10.** Phylogenetic relationships of common buzzard MHC-IIB exon 2 alleles across closely related Afroaves species from ML-based phylogenies. **Figure S11. **Phylogenetic relationships of common buzzard MHC-I exon 2 and MHC-IIA exon 2 alleles among other avian orders from ML-based phylogenies.

## Data Availability

The genotype dataset generated and analyzed during the current study and genomic data for the MHC region is available in the figshare repository (https://doi.org/10.6084/m9.figshare.16885255.v1). Metadata and MHC transcript sequences can be found on the figshare repository (https://doi.org/10.6084/m9.figshare.18282818.v1). Sequences analyzed during the current study have been deposited to NCBI GenBank (http://www.ncbi.nlm.nih.gov/genbank) under the GenBank accession #s: OL311188‒OL311318, OP490257‒OP490259, OP503112‒OP503121, OQ390037, OQ414190‒OQ414202, OQ428163‒OQ428174.

## Abbreviations

CNV: Copy number variation
dN: Rate of nonsynonymous substitutions
dS: Rate of synonymous substitutions
EPS: Episodic positive selection
Hi-C: High-throughput chromosome conformation capture
HiFi: High-Fidelity
HLA: Human leukocyte antigen
MHC: Major histocompatibility complex
MHC-I: MHC class I
MHC-II: MHC class II
ML: Maximum likelihood
PBR: Peptide binding residues
PNS: Pervasive negative selection
PPS: Pervasive positive selection
PSS: Positively selected sites
RNAseq: RNA sequencing
TSP: Trans-species polymorphism
