# Supplementary material for: High functional allelic diversity and copy number in both MHC classes in the common buzzard
Source: BMC Ecol Evol. 2023 Jun 24;23:24. doi: 10.1186/s12862-023-02135-9 (PMC10290333; doi:10.1186/s12862-023-02135-9)
Supplement: Supplementary file 1 — Additional file 1: Table S1. AmpliSAS parameter settings. Table S2. Recombination signal at MHC class I exon 3 and MHC class II exon 2 of common buzzards. Table S3. Confirmed MHC haplotypes based on long-read sequencing in common buzzards. Table S4. RNA transcript data confirms all three MHC loci are expressed. Table S5. HLA sequences used for Table 3. Figure S1. Common buzzard MHC class I exon 3 alleles translation with evolutionarily conserved sites. Figure S2. Common buzzard MHC class I exon 3 allele count. Figure S3. Common buzzard MHC class IIB exon 2 alleles translation with evolutionarily conserved sites. Figure S4. Common buzzard MHC class IIB exon 2 allele count. Figure S5. Common buzzard MHC loci copy number inferred from long-read sequencing. Figure S6. Alignments of amino acid sequences for MHC class I exon 2 and MHC class IIA exon 2 of common buzzards. Figure S7. Phylogenetic relationships of common buzzard MHC-I exon 3 alleles across closely related Afroaves species. Figure S8. Phylogenetic relationships of common buzzard MHC-IIB exon 2 alleles across closely related Afroaves species. Figure S9. Phylogenetic relationships of common buzzard MHC-I exon 3 alleles across closely related Afroaves species from ML-based phylogenies. Figure S10. Phylogenetic relationships of common buzzard MHC-IIB exon 2 alleles across closely related Afroaves species from ML-based phylogenies. Figure S11. Phylogenetic relationships of common buzzard MHC-I exon 2 and MHC-IIA exon 2 alleles among other avian orders from ML-based phylogenies. [file 12862_2023_2135_MOESM1_ESM.pdf]

BMC Ecology and Evolution

High functional allelic diversity and copy number in both MHC classes in the common buzzard

J. Winternitz✉, N. Chakarov, T. Rinaud, M. Ottensmann, O. Krüger

✉ Jamie C. Winternitz
Department of Animal Behaviour, Bielefeld University, Bielefeld 33615, Germany
Email: jcwinternitz@gmail.com

SUPPLEMENTARY MATERIAL

Supplementary Tables **2**

Table S1. AmpliSAS parameter settings. 2
Table S2. Recombination signal at MHC class I exon 3 and MHC class IIB exon 2 of common buzzards. 2
Table S3. Confirmed MHC haplotypes based on long-read sequencing in common buzzards. 3
Table S4. RNA transcript data confirms all three MHC loci are expressed. 5
Table S5. HLA sequences used for Table 3. 6

Supplementary Figures **9**

Figure S1. Common buzzard MHC class I exon 3 alleles translation with evolutionarily conserved sites 9
Figure S2. Common buzzard MHC class I exon 3 allele count. 11
Figure S3. Common buzzard MHC class IIB exon 2 alleles translation with evolutionarily conserved sites 12
Figure S4. Common buzzard MHC class I exon 3 allele count. 13
Figure S5. Common buzzard MHC loci copy number inferred from long-read sequencing. 14
Figure S6. Alignments of amino acid sequences for MHC class I exon 2 and MHC class IIA exon 2 of common buzzards. 15
Figure S7. Phylogenetic relationships of common buzzard MHC-I exon 3 alleles across closely related Afroaves species 16
Figure S8. Phylogenetic relationships of common buzzard MHC-IIB exon 2 alleles across closely related Afroaves species 17
Figure S9. Phylogenetic relationships of common buzzard MHC-I exon 3 alleles across closely related Afroaves species from ML-based phylogenies. 18
Figure S10. Phylogenetic relationships of common buzzard MHC-IIB exon 2 alleles across closely related Afroaves species from ML-based phylogenies. 19
Figure S11. Phylogenetic relationships of common buzzard MHC-I exon 2 and MHC-IIA exon 2 alleles among other avian orders from ML-based phylogenies. 20

References **21**

Supplementary Tables

Table S1. AmpliSAS parameter settings.

Parameter settings for AmpliSAS genotyping of merged and cleaned reads for *Buteo buteo* MHC class I exon 3 (MHC-I) and MHC class IIB exon 2 (MHC-II). The exact length for MHC-I was set to 262bp.

parameter	value for MHC-I	value for MHC-II
substitution_threshold	0.8	1
indel_threshold	0.001	0.001
cluster_exact_length	1	.
cluster_inframe	1	1
min_dominant_frequency_threshold	.	50
min_amplicon_depth	100	100
min_amplicon_seq_frequency	3	4
discard_frameshifts	1	1
max_amplicon_length_error	.	4
min_chimera_length	10	10
max_allele_number	6	6

Table S2. Recombination signal at MHC class I exon 3 and MHC class IIB exon 2 of common buzzards.

Recombination events were identified with the following methods using RPD4 v.4.101 software [1]: R: RDP, G: GENECONV, M: Maxchi, C: Chimaera, S: SiScan, T: 3Seq. n_{seq}: number of sequences, n_{sites}: number of nucleotide sites, $n_{recombinant\ sequences}$: number of recombinant sequences inferred. Accepted recombination events are in bold. A recombination event was recognized when supported by two or more algorithms. Next, we proceeded with manual examination. Briefly, we sequentially examined all detected recombination events for the following: the breakpoint positions and the identity of the recombinants could be verified, the recombination event was detected in more than one sequence, the average p-value for the method detecting recombination was less than 0.05, and there was no warning that the apparent recombination signal could have been caused by an evolutionary process other than recombination. If all these criteria were met then the recombination event was accepted, if not, the event was rejected.

MHC class and exon	n_{seq} (n_{sites})	$n_{recombinant\ sequences}$	Beginning breakpoint	Ending breakpoint	Methods (avg. p-val)
MHC-I exon 2	10 (264)	1	54	105	M (0.002), C (0.004), T (0.003)
MHC-I exon 3	91 (262)	9	5	83	M (0.995), T (0.004)
		5	21	133	M (0.809), T(0.030)
MHC-IIB exon 2	41 (258)	**13**	**128**	**257**	**M (0.001), C (0.011), S (0.001), T (<0.0001)**
		**2**	**182**	**254**	**M (<0.0001), C (0.0001), T (<0.0001)**
		1	80	181	M (0.004), T (0.033)
		8	2	108	M (0.313), S (0.027)
		**9**	**53**	**158**	**R (0.999), G (0.130), M (0.028), S (0.042)**

Table S3. Confirmed MHC haplotypes based on long-read sequencing in common buzzards.

To call haplotypes, we performed a series of steps starting with PacBio HiFi long reads (mean coverage of 14 reads per individual). MHC regions were identified via BLAST of multiple known avian MHC-I and II loci (AB119993, AB872442, CHKMHBFVB, CHKMHCBCHA, EF370956, EU442606, HM008713, HM008714, HM008715, KC282841, KC282842, KC282843, KC282844, KP182409, KY511591, KY511592, NM_001310349, NM_001245061, MN061408, MN061399, MN061393, MK981897, MK981896, MK829176, KF041454, HQ203731). Reads matching MHC regions were then de novo assembled into haplotype-aware contigs with Phasebook [2] using the parameters -t 8 -p hifi -g small -x -min_cov 2 -min_cluster 2 -min_allele_cov 2. For genomic context, the resulting contigs were aligned to the MHC region (96,746 bp) of Hi-C generated chromosome 29 of three common buzzards (*Buteo buteo*) using Minimap2 [3] with the preset PacBio settings. Haplotype-aware contigs that overlapped MHC-I and MHC-II annotated regions of ch29 were then blasted to all 91 MHC-I exon 3 alleles and 41 MHC-IIB exon 2 alleles to determine the alleles present at each locus. GenBank accession numbers for full-length or nearly full-length alleles appear under the respective locus for each individual. The 4th exon 2 at MHC class IIB identified in one individual contig (1060) based on Flye assembly did not match with any NGS amplicon sequenced allele. This could be due to an error in Flye assembly or to the relatively more conservative nature of Phasebook haplotype-aware assembly. Column names for each MHC class show the putative locus names and the location on chromosome 29 of the collapsed haplotype of individual HiFi ID 263 (GenBank accension # OQ390037).

Samples			MHC class I exon 2 & 3					
HiFi ID	Contig ID	Hap#	UAA exon 2 (470,791-471,054)	UAA exon 3 (471,810-472,085)	UBA exon 2 (476,781-477,044)	UBA exon 3 (478,256-478,531)	UCA exon 2 (500,791-500,528)	UCA exon 3 (499,780-499,505)
48	1271	1	Bubute-N*10<br>OQ428174	Bubute-N*01	Bubute-N*09<br>OQ428169	Bubute-N*19		
		2						
64	3011	1	Bubute-N*10<br>OQ428173	Bubute-N*01	Bubute-N*07<br>OQ428168	Bubute-N*30	Bubute-N*07<br>OQ428164	Bubute-N*04
		2			Bubute-N*08		Bubute-N*08	
263	1827	1	Bubute-N*06<br>OQ428171	Bubute-N*01	Bubute-N*05<br>OQ428167	Bubute-N*13	Bubute-N*04<br>OQ428163	Bubute-N*14
		2						
326	3164	1	Bubute-N*10<br>OQ428172	Bubute-N*01	Bubute-N*02<br>OQ428166	Bubute-N*05		
		2	Bubute-N*03<br>OQ428170	Bubute-N*01	Bubute-N*01<br>OQ428165	Bubute-N*03		

Samples			MHC class IIA & IIB exon 2					
HiFi ID	Contig ID	Hap#	DRA1 (421,667-421,410)	DRB1 (423,685-423,954)	DRA2 (427,067-426,810)	DRB2 (429,085-429,354)	DRA3 (432,467-432,210)	DRB3 (434,418-434,687)
48	1271	1	Bubute-DRA*01<br>OQ414192	Bubute-DRB*26<br>OL311304	Bubute-DRA*01<br>OQ414194			
		2						
64	2574	1				Bubute-DRB*12<br>OL311290	Bubute-DRA*01<br>OQ414196	Butbut-DAB*01<br>OQ414202
		2	Bubute-DRA*01<br>OQ414191	Bubute-DRB*27<br>OL311305			Bubute-DRA*02<br>OQ414197	Bubute-DRB*16<br>OL311294
263	2549	1	Bubute-DRA*03<br>OP490259	Bubute-DRB*01<br>OQ414199			Bubute-DRA*02<br>OQ414197	Bubute-DRB*04<br>OQ414201
		2						
326	1060	1	Bubute-DRA*02<br>OQ414190	Bubute-DRB*09<br>OQ414198	Bubute-DRA*01<br>OQ414193			Bubute-DRB*04<br>OQ414200
		2		Bubute-DRB*01<br>OL311287	Bubute-DRA*02<br>OQ414195	Bubute-DRB*14<br>OL311292	Bubute-DRA*02<br>OQ414197	Bubute-DRB*04<br>OQ414200

Table S4. RNA transcript data confirms all three MHC loci are expressed.

Alleles identified from haplotype-aware contigs were blast-searched against RNA transcripts from 81 individuals obtained for a different study. Presence of these sequences in transcript data combined with their genomic location (locus number) confirmed that all three loci have alleles found to be expressed.

MHC class and exon	Allele ID	Locus number	Expressed
MHC-I exon 2	Bubute-N*03	1	1
MHC-I exon 2	Bubute-N*06	1	1
MHC-I exon 2	Bubute-N*10	1	1
MHC-I exon 2	Bubute-N*01	2	0
MHC-I exon 2	Bubute-N*02	2	1
MHC-I exon 2	Bubute-N*05	2	1
MHC-I exon 2	Bubute-N*09	2	0
MHC-I exon 2	Bubute-N*04	3	0
MHC-I exon 2	Bubute-N*07	2, 3	0
MHC-I exon 2	Bubute-N*08	2, 3	1
MHC-I exon 3	Bubute-N*01	1	1
MHC-I exon 3	Bubute-N*03	2	1
MHC-I exon 3	Bubute-N*05	2	1
MHC-I exon 3	Bubute-N*13	2	1
MHC-I exon 3	Bubute-N*19	2	0
MHC-I exon 3	Bubute-N*30	2	0
MHC-I exon 3	Bubute-N*04	3	1
MHC-I exon 3	Bubute-N*14	3	1
MHC-IIA exon 2	Bubute-DRA*01	1,2,3	1
MHC-IIA exon 2	Bubute-DRA*02	1,2,3	1
MHC-IIA exon 2	Bubute-DRA*03	1	1
MHC-IIB exon 2	Bubute-DRB*01	1	1
MHC-IIB exon 2	Bubute-DRB*09	1	1
MHC-IIB exon 2	Bubute-DRB*27	1	1
MHC-IIB exon 2	Bubute-DRB*26	1	1
MHC-IIB exon 2	Bubute-DRB*12	2	1
MHC-IIB exon 2	Bubute-DRB*14	2	1
MHC-IIB exon 2	Butbut-DAB*01	3	1
MHC-IIB exon 2	Bubute-DRB*04	3	1
MHC-IIB exon 2	Bubute-DRB*16	3	0

Table S5. HLA sequences used for Table 3.

Human MHC class I HLA-A, B, C, and class II HLA-DRB1 alleles from the European population classified as "common" in the Common and Well-Documented allele catalog 3.0.0 [4] as well as HLA-DRA*01:01 from the monomorphic DRA locus were downloaded from IPD-IMGT/HLA Database version 3.51 [5].

IPD-IMGT accession	Allele name	Locus
HLA00001	A*01:01:01:01	A
HLA00002	A*01:02:01:01	A
HLA00037	A*03:01:01:01	A
HLA00040	A*03:02:01:01	A
HLA00043	A*11:01:01:01	A
HLA00116	A*68:01:02:01	A
HLA05918	A*68:01:01:02	A
HLA00117	A*68:02:01:01	A
HLA00073	A*26:01:01:01	A
HLA00080	A*26:08:01:01	A
HLA00109	A*34:02:01:01	A
HLA00085	A*29:01:01:01	A
HLA00086	A*29:02:01:01	A
HLA00097	A*31:01:02:01	A
HLA00971	A*33:05	A
HLA00129	A*74:03:01:01	A
HLA00101	A*32:01:01:01	A
HLA00089	A*30:01:01:01	A
HLA00090	A*30:02:01:01	A
HLA00092	A*30:04:01:01	A
HLA00013	A*02:08:01	A
HLA00026	A*02:20:01	A
HLA01035	A*02:35:01	A
HLA00050	A*24:02:01:01	A
HLA00132	B*07:02:01:01	B
HLA00142	B*07:10	B
HLA00136	B*07:04:01	B
HLA00315	B*42:01:01:01	B
HLA00146	B*08:01:01:01	B
HLA02219	B*08:01:02	B
HLA00312	B*41:01:01:01	B
HLA00313	B*41:02:01:01	B
HLA00329	B*45:01:01:01	B
HLA00342	B*50:02:01:01	B
HLA00158	B*14:02:01:01	B
HLA01221	B*39:24:01	B
HLA00173	B*15:10:01:01	B
HLA00162	B*15:01:01:01	B
HLA00202	B*15:39:01:01	B

HLA00171	B*15:08:01:01	B
HLA00187	B*15:24:01	B
HLA00213	B*18:01:01:01	B
HLA00215	B*18:03:01:01	B
HLA00180	B*15:17:01:01	B
HLA00386	B*58:01:01:01	B
HLA00382	B*57:02:01:01	B
HLA00344	B*51:01:01:01	B
HLA00352	B*51:07:01	B
HLA00354	B*51:09:01	B
HLA00350	B*51:05:01:01	B
HLA00353	B*51:08:01:01	B
HLA00364	B*53:01:01:01	B
HLA00368	B*55:01:01:01	B
HLA00318	B*44:02:01:01	B
HLA00322	B*44:05:01:01	B
HLA00319	B*44:03:01:01	B
HLA00320	B*44:03:02:01	B
HLA00321	B*44:04	B
HLA00222	B*27:03	B
HLA00226	B*27:05:03	B
HLA00230	B*27:09	B
HLA00235	B*27:14	B
HLA00392	B*73:01:01:01	B
HLA00405	C*02:02:02:01	C
HLA00467	C*15:02:01:01	C
HLA00471	C*15:05:02:01	C
HLA00469	C*15:04:01:01	C
HLA00472	C*15:06:01:01	C
HLA00427	C*05:01:01:01	C
HLA00446	C*08:02:01:01	C
HLA00455	C*12:03:01:01	C
HLA00476	C*16:02:01:01	C
HLA00430	C*06:02:01:01	C
HLA00462	C*14:02:01:01	C
HLA00464	C*14:03:01:01	C
HLA00420	C*04:01:01:01	C
HLA00423	C*04:03:01:01	C
HLA00411	C*03:03:01:01	C
HLA00414	C*03:04:02:01	C
HLA00433	C*07:01:01:01	C
HLA00434	C*07:02:01:01	C
HLA00436	C*07:04:01:01	C
HLA14101	C*17:01:01:05	C
HLA00662	DRA*01:01:01:01	DRA
HLA00687	DRB1*04:02:01	DRB1
HLA00689	DRB1*04:04:01:01	DRB1

HLA00694	DRB1*04:08:01:01	DRB1
HLA02172	DRB1*04:06:02	DRB1
HLA00671	DRB1*03:01:01:01	DRB1
HLA00676	DRB1*03:04:01	DRB1
HLA00673	DRB1*03:02:01:01	DRB1
HLA00752	DRB1*11:01:02:01	DRB1
HLA03812	DRB1*11:01:08	DRB1
HLA00766	DRB1*11:12:01	DRB1
HLA00769	DRB1*11:15:01:01	DRB1
HLA00754	DRB1*11:02:01:01	DRB1
HLA00755	DRB1*11:03:01	DRB1
HLA00756	DRB1*11:04:01:01	DRB1
HLA00802	DRB1*13:05:01:01	DRB1
HLA00813	DRB1*13:15	DRB1
HLA00838	DRB1*14:06:01	DRB1
HLA00799	DRB1*13:03:01:01	DRB1
HLA00723	DRB1*08:01:01:01	DRB1
HLA00728	DRB1*08:04:01:01	DRB1
HLA00732	DRB1*08:06:01:01	DRB1
HLA00833	DRB1*14:01:01:01	DRB1
HLA00839	DRB1*14:07:01	DRB1
HLA00836	DRB1*14:04:01:01	DRB1
HLA00664	DRB1*01:01:01:01	DRB1
HLA00665	DRB1*01:02:01:01	DRB1
HLA00667	DRB1*01:03:01:01	DRB1
HLA03453	DRB1*15:01:01:02	DRB1
HLA00876	DRB1*16:01:01:01	DRB1

Supplementary Figures

Figure S1. Common buzzard MHC class I exon 3 alleles translation with evolutionarily conserved sites.

Alignment of MHC-I exon 3 from human and white-tailed eagle (*Haliaeetus albicilla*) sequences with *Buteo buteo* putative alleles (numbering of residues 92-178 based on HLA-A alignment). Identity, representing the pairwise % identity at individual sites, is shown above the alignment. Dots indicate amino acids identical to the human HLA-A reference. Sites in disagreement to the reference are highlighted. The numbers in blue at specific sites above the alignment indicate evolutionarily well conserved sites that are features of classical MHC-I loci [6, 7]. These include the following:

i) Highly conserved amino acid residues that bind the N- (Y159, Y171) and C- (T143, K146, W147) termini of the peptide to anchor it.

ii) Cysteine (C) residues that form the disulphide bonds and turns in the alpha2 domain (exon 3) (C101-C164, G112).

iii) Conserved salt bridges (H93-D119).

iv) TCR contact sites constant across all HLA class I A, B, and C loci (K146, E154, Q155, R157, Y159, G162, R170).

v) Peptide binding sites (D119, T143, K146, W147, Y159, L160, Y171).

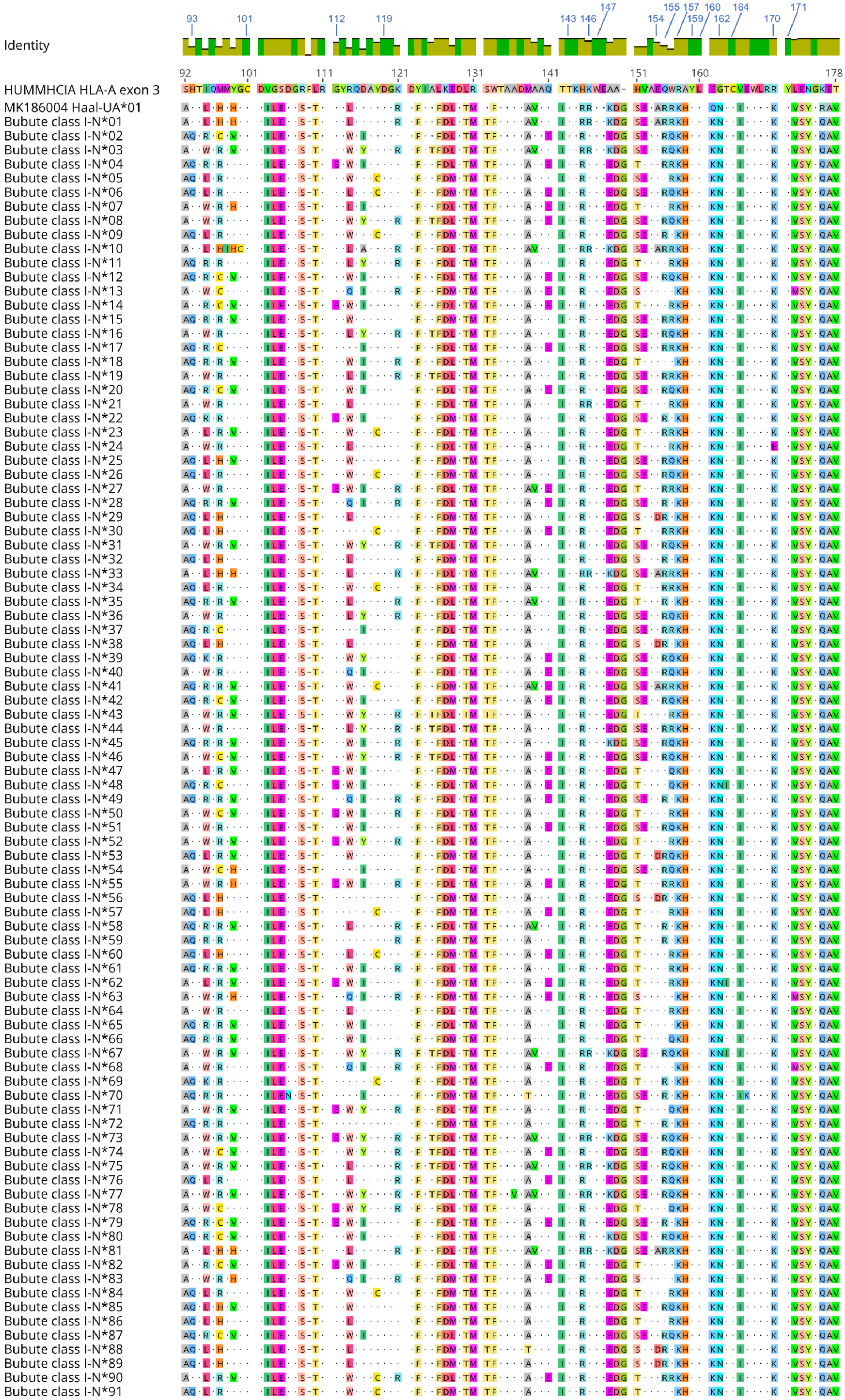

Identity
93 101 112 119 143 146 147 154 155 157 159 160 162 164 170 171
92 101 111 121 131 141 151 160 178
HUMMHCIA HLA-A exon 3
MK186004 Haal-UA*01
Bubute class I-N*01
Bubute class I-N*02
Bubute class I-N*03
Bubute class I-N*04
Bubute class I-N*05
Bubute class I-N*06
Bubute class I-N*07
Bubute class I-N*08
Bubute class I-N*09
Bubute class I-N*10
Bubute class I-N*11
Bubute class I-N*12
Bubute class I-N*13
Bubute class I-N*14
Bubute class I-N*15
Bubute class I-N*16
Bubute class I-N*17
Bubute class I-N*18
Bubute class I-N*19
Bubute class I-N*20
Bubute class I-N*21
Bubute class I-N*22
Bubute class I-N*23
Bubute class I-N*24
Bubute class I-N*25
Bubute class I-N*26
Bubute class I-N*27
Bubute class I-N*28
Bubute class I-N*29
Bubute class I-N*30
Bubute class I-N*31
Bubute class I-N*32
Bubute class I-N*33
Bubute class I-N*34
Bubute class I-N*35
Bubute class I-N*36
Bubute class I-N*37
Bubute class I-N*38
Bubute class I-N*39
Bubute class I-N*40
Bubute class I-N*41
Bubute class I-N*42
Bubute class I-N*43
Bubute class I-N*44
Bubute class I-N*45
Bubute class I-N*46
Bubute class I-N*47
Bubute class I-N*48
Bubute class I-N*49
Bubute class I-N*50
Bubute class I-N*51
Bubute class I-N*52
Bubute class I-N*53
Bubute class I-N*54
Bubute class I-N*55
Bubute class I-N*56
Bubute class I-N*57
Bubute class I-N*58
Bubute class I-N*59
Bubute class I-N*60
Bubute class I-N*61
Bubute class I-N*62
Bubute class I-N*63
Bubute class I-N*64
Bubute class I-N*65
Bubute class I-N*66
Bubute class I-N*67
Bubute class I-N*68
Bubute class I-N*69
Bubute class I-N*70
Bubute class I-N*71
Bubute class I-N*72
Bubute class I-N*73
Bubute class I-N*74
Bubute class I-N*75
Bubute class I-N*76
Bubute class I-N*77
Bubute class I-N*78
Bubute class I-N*79
Bubute class I-N*80
Bubute class I-N*81
Bubute class I-N*82
Bubute class I-N*83
Bubute class I-N*84
Bubute class I-N*85
Bubute class I-N*86
Bubute class I-N*87
Bubute class I-N*88
Bubute class I-N*89
Bubute class I-N*90
Bubute class I-N*91

Figure S2. Common buzzard MHC class I exon 3 allele count.

a) Common buzzard MHC-I exon 3 allele count per amplicon shows no statistically significant relationship with (log) read depth, indicating our allele discovery and final genotypes were unbiased by amplicon read coverage. Summary of results from the linear regression model: $R^2 = 0.003$, $F(1,111) = 0.277$, $p = 0.600$. b) Histogram of alleles per individual in the population. The range of alleles per genotype was 1 to 8, and the average was 4.81.

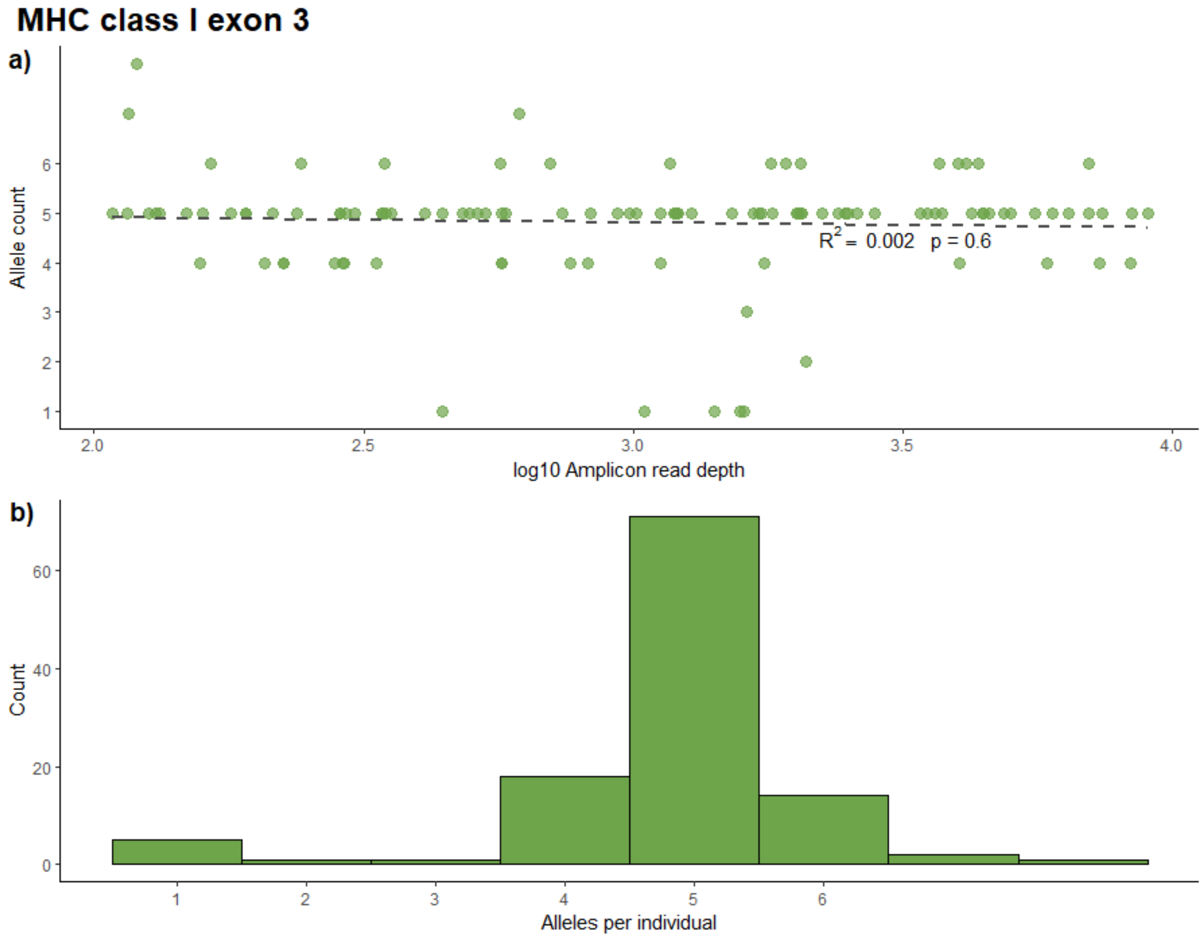

Figure S3. Common buzzard MHC class IIB exon 2 alleles translation with evolutionarily conserved sites.

Alignment of human MHC-II HLA-DRB exon 2 (residues 1-95) with *Buteo buteo* putative MHC-IIB partial exon 2 alleles. Identity, representing the pairwise % identity at individual sites, is shown above the alignment. Dots indicate amino acids identical to the human HLA-DRB reference. Sites in disagreement to the reference are highlighted. The numbers in blue at specific sites indicate evolutionarily well conserved sites that are features of classical MHC-II loci [6, 7]. These include the following:

i) Highly conserved amino acid residues that bind the peptide to anchor it (W61, H81, N82).

ii) Cysteine (C) residues that form the disulphide bonds and turns (C15-C79, G45, G54).

iii) Glycosylation sites (N19, T21).

iv) TCR contact sites (E69, R72, D76, H81).

v) Unassigned sites (F/Y40, D/N41, S42, A58, N62, L68).

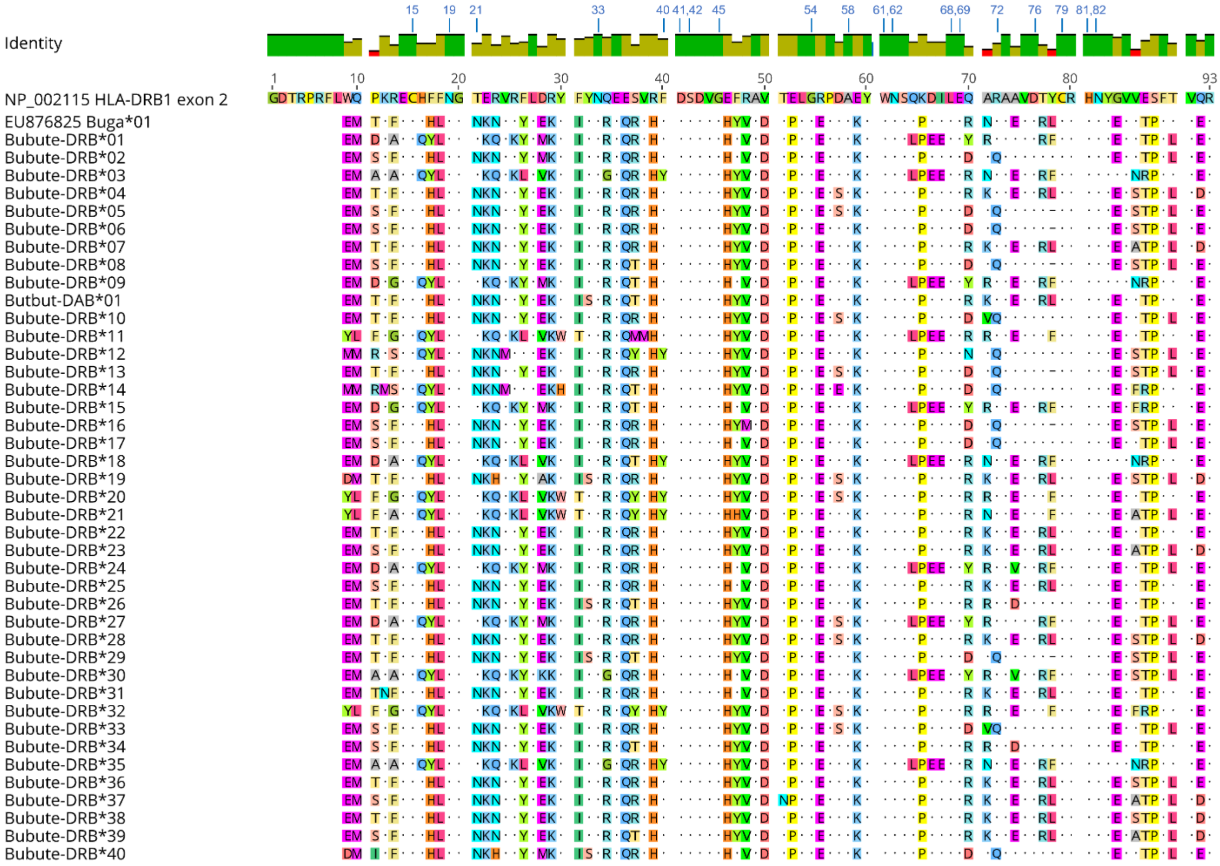

Figure S4. Common buzzard MHC class I exon 3 allele count.

a) Common buzzard MHC-IIB exon 2 allele count per amplicon shows no statistically significant relationship with (log) read depth, indicating our allele discovery and final genotypes were unbiased by amplicon read coverage. Summary of results from the linear regression model: $R^2 = 0.018$, $F(1,123) = 2.226$, $p = 0.138$. b) Histogram of alleles per individual in the population. The range of alleles per genotype was 2 to 5, and the average was 3.65.

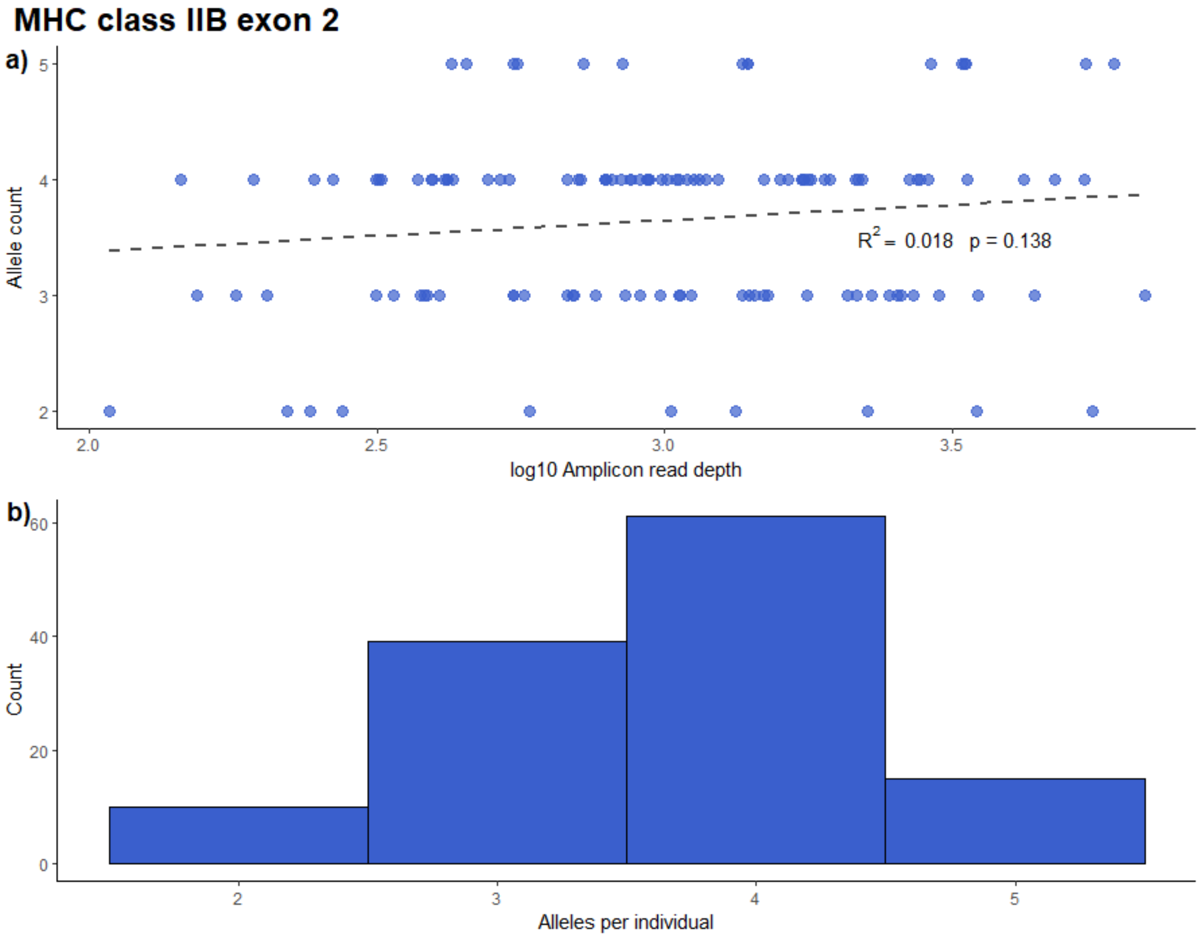

Figure S5. Common buzzard MHC loci copy number inferred from long-read sequencing.

a) MHC region of chromosome 29 from a consensus of three common buzzards (*Buteo buteo*). Positions of class I exon 2 (light green) exon 3 (green) and class IIA exon 2 (light blue) and IIB exon 2 (blue) are indicated by colored triangles. Both b) MHC-I and c) MHC-II regions mapped to scaffold 33, representing chromosome 29 (1,742,570 bp), using Minimap2 [3] in Geneious Prime 2022.2.1 (https://www.geneious.com) (with options -x map-pb –frag=yes –secondary=no -a). This allowed us to confirm that MHC class I exon 3 and class IIB exon 2 are present in at least three locations in *Buteo buteo* ch29, and that copy number variation is demonstrated for MHC-IIB. Numbers above the HiC scaffold 33 and Consensus sequence indicate base pair positions along ch29 and along the visible sequence region, respectively. The filled star indicates the locus matching Bubute class I-N*01. The contig sequence that maps to the HiC scaffold reference genome (highlighted yellow) is in black and unaligned parts of the contig sequence are in light grey. Suffix numbers at the end of contigs indicate the individual ID for genomic sequencing. Rev: reverse orientation.

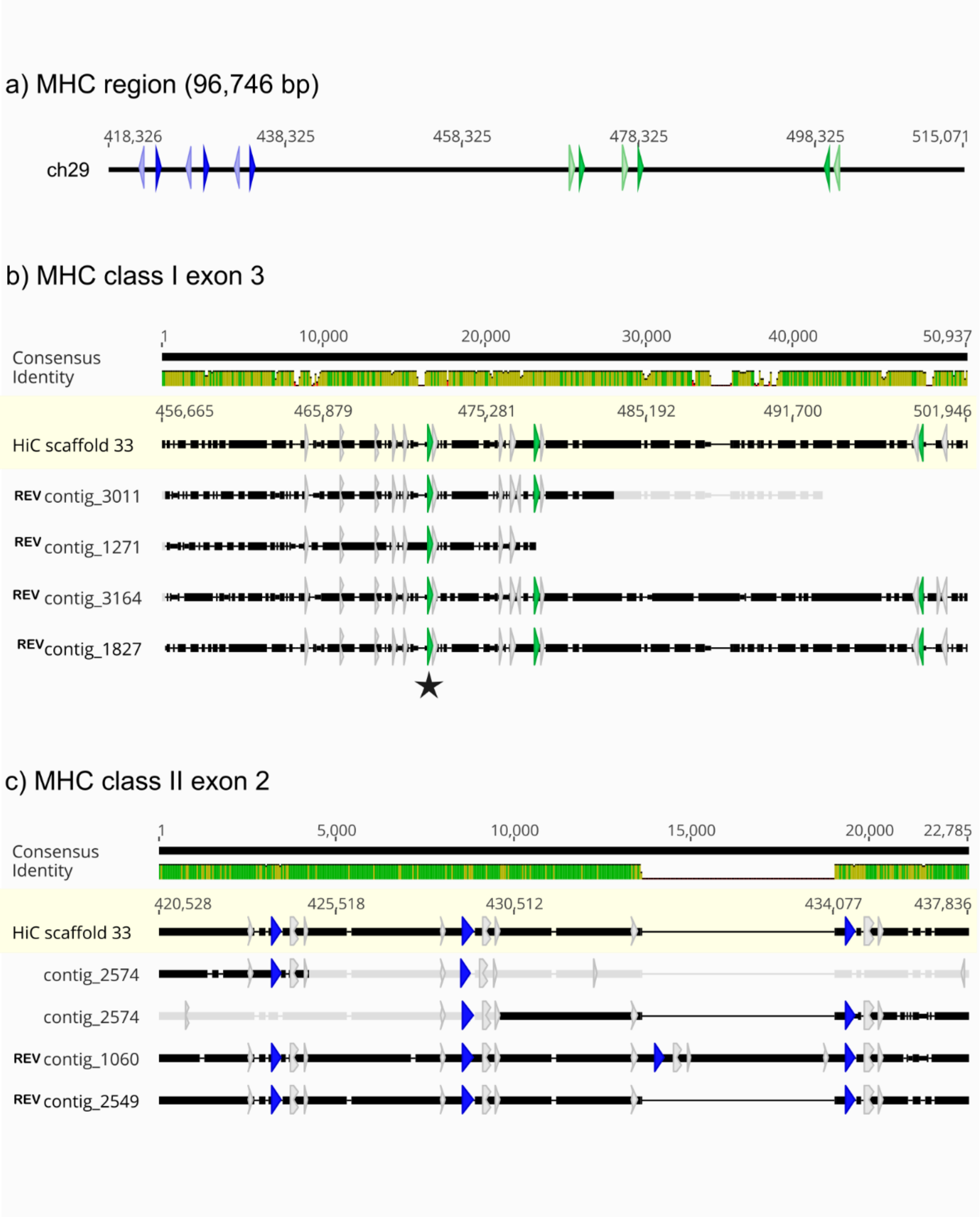

Figure S6. Alignments of amino acid sequences for MHC class I exon 2 and MHC class IIA exon 2 of common buzzards.

a) Alignment of *Buteo buteo* MHC-I exon 2 putative alleles include sequences from human (accession # L06425) and chicken (accession # KF032302), with numbering of residues 2-90 based on HLA-A alignment (2-89). Dots indicate amino acids identical to the top sequence of MHC-I exon 2 and MHC-IIA exon 2, respectively. Circles represent human peptide binding residues (PBR) from [8] and squares represent chicken PBR from [9]. PPS: pervasive positive selection, EPS: episodic positive selection, PNS: pervasive negative selection. b) Alignment of *Buteo buteo* MHC-IIA exon 2 putative alleles include a sequence from chicken (accession # HQ203731), with numbering of residues 4-88 based on chicken BL-A alignment. We did not infer sites under selection for MHC-IIA exon 2 because we had too few sequences for selection analysis to be meaningful. Circles represent human PBR from [10].

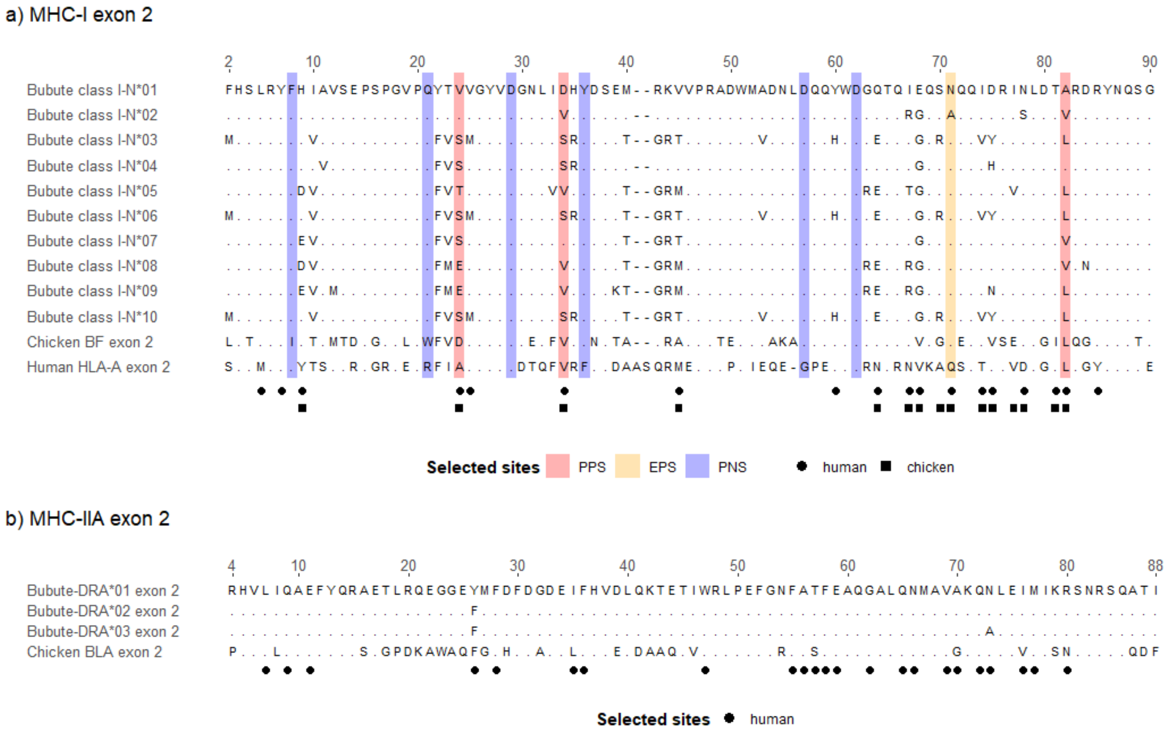

Figure S7. Phylogenetic relationships of common buzzard MHC-I exon 3 alleles across closely related Afroaves species.

Approximately-maximum-likelihood phylogenetic reconstruction for 91 *Buteo buteo* alleles of MHC class I exon 3. Scale bars indicate the number of substitutions per site. Values of clades with > 0.8 bootstrap support are shown. Alleles do not fall neatly into three clades, as would be expected for three locus copies that evolved independently and for long enough to differentiate from each other. This suggests that either common buzzard alleles have not evolved independently and may be shared across loci or have undergone recombination (gene conversion), or that gene duplication events are very recent and have not had enough time to diversify. Common buzzard MHC-I exon 3 alleles mostly clustered with the closest related species in our tree separated by 23.8 myr, the white-tailed eagle (*Haliaeetus albicilla*). Common buzzard allele Bubute class I-N*01, which was found in every genotyped individual and is likely fixed at a locus, was in the clade that included the allele Haal-UA*02 exon 3 (Accession # MK186005) of the white-tailed eagle.

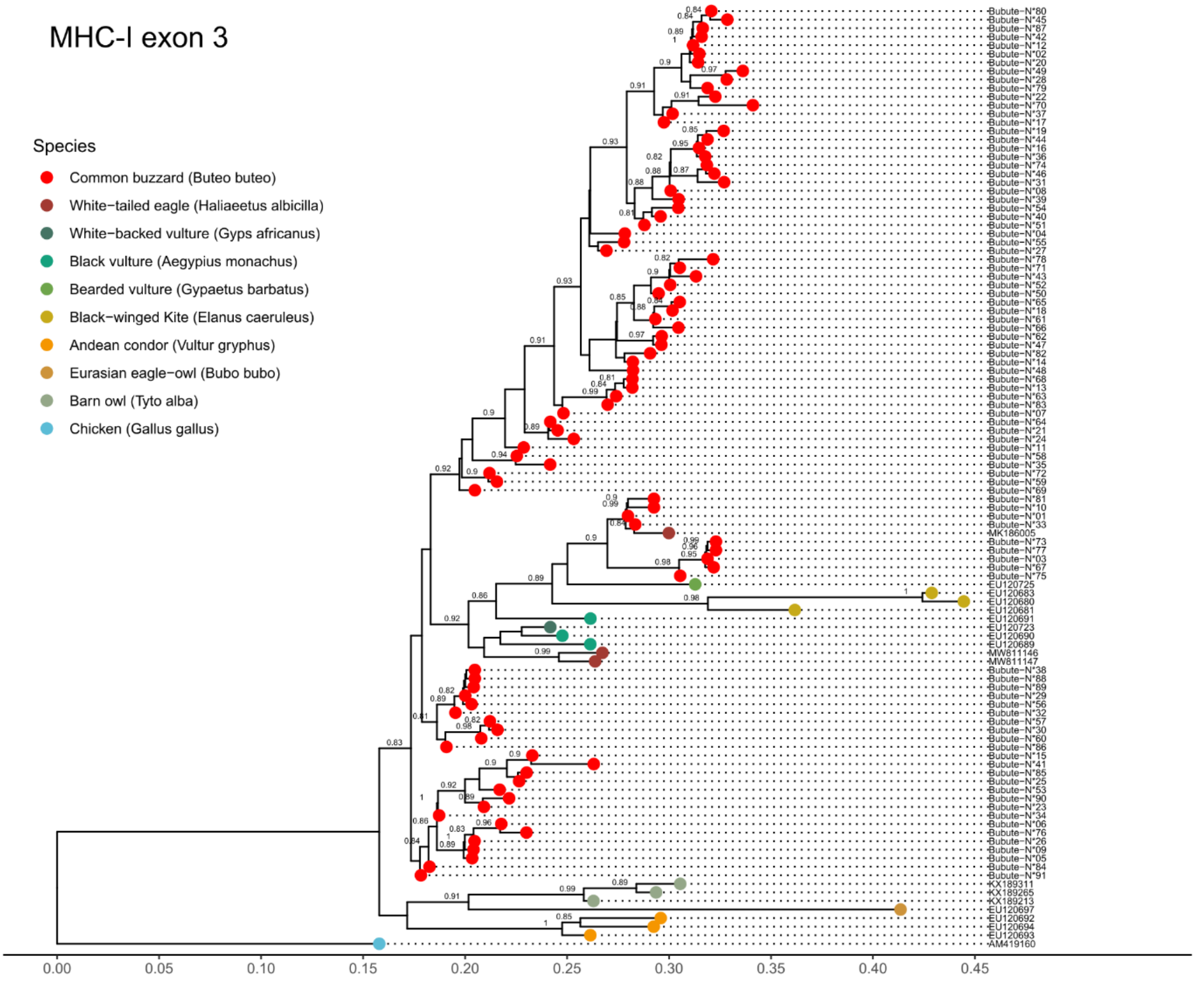

Figure S8. Phylogenetic relationships of common buzzard MHC-IIB exon 2 alleles across closely related Afroaves species.

Approximately-maximum-likelihood phylogenetic reconstruction for 41 *Buteo buteo* alleles of MHC class IIB exon 2. Scale bars indicate the number of substitutions per site. Values of clades with > 0.8 bootstrap support are shown. As with Figure S6, alleles do not fall neatly into three clades, as would be expected for three locus copies that evolved independently and for long enough to differentiate from each other. This suggests that either common buzzard alleles have not evolved independently and may be shared across loci or have undergone recombination (gene conversion), or that gene duplication events are very recent and have not had enough time to diversify. Common buzzard MHC-IIB exon 2 alleles clustered both within and between species, including distantly related species separated 35.1 myr, the white-backed vulture (*Gyps africanus*) and black vulture (*Aegypius monachus*).

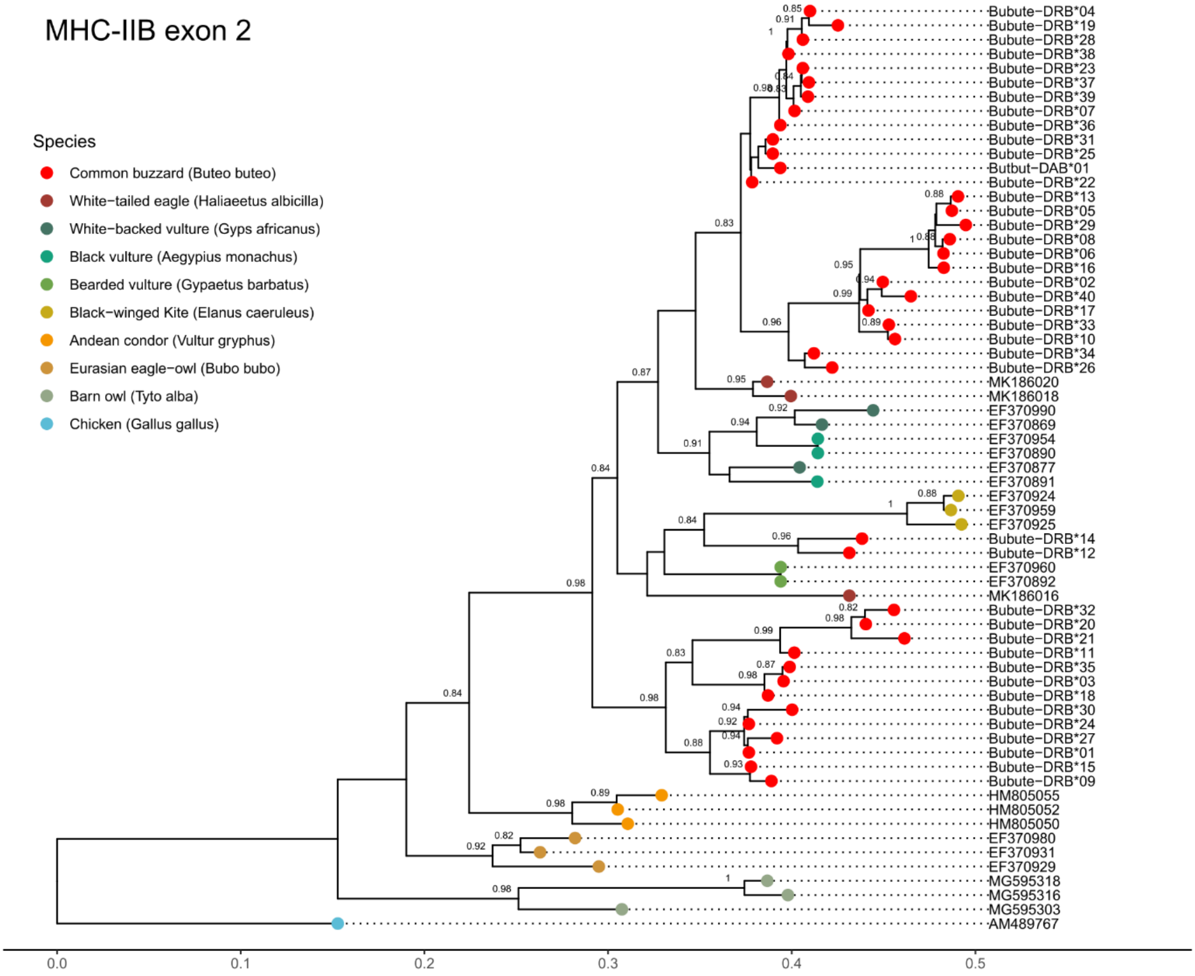

Figure S9. Phylogenetic relationships of common buzzard MHC-I exon 3 alleles across closely related Afroaves species from ML-based phylogenies.

Maximum-likelihood phylogenetic reconstruction for 91 *Buteo buteo* alleles of MHC class I exon 3 was performed using IQ-TREE [11]. We used ModelFinder [12] implemented within IQ-TREE web server [13] to select the best substitution model based on Bayesian information criterion (BIC). Branch support was assessed using 1000 ultrafast bootstrap replicates (UFBoot [14]), using the K2P+R4 model. Scale bars indicate the number of substitutions per site. Values of clades with > 70 bootstrap support are shown. MHC-I appears to have an ancient Locus 1 lineage (containing sequence Bubute-N*01) shared within the order Accipitriformes with white-tailed eagles (*Haliaeetus albicilla).*

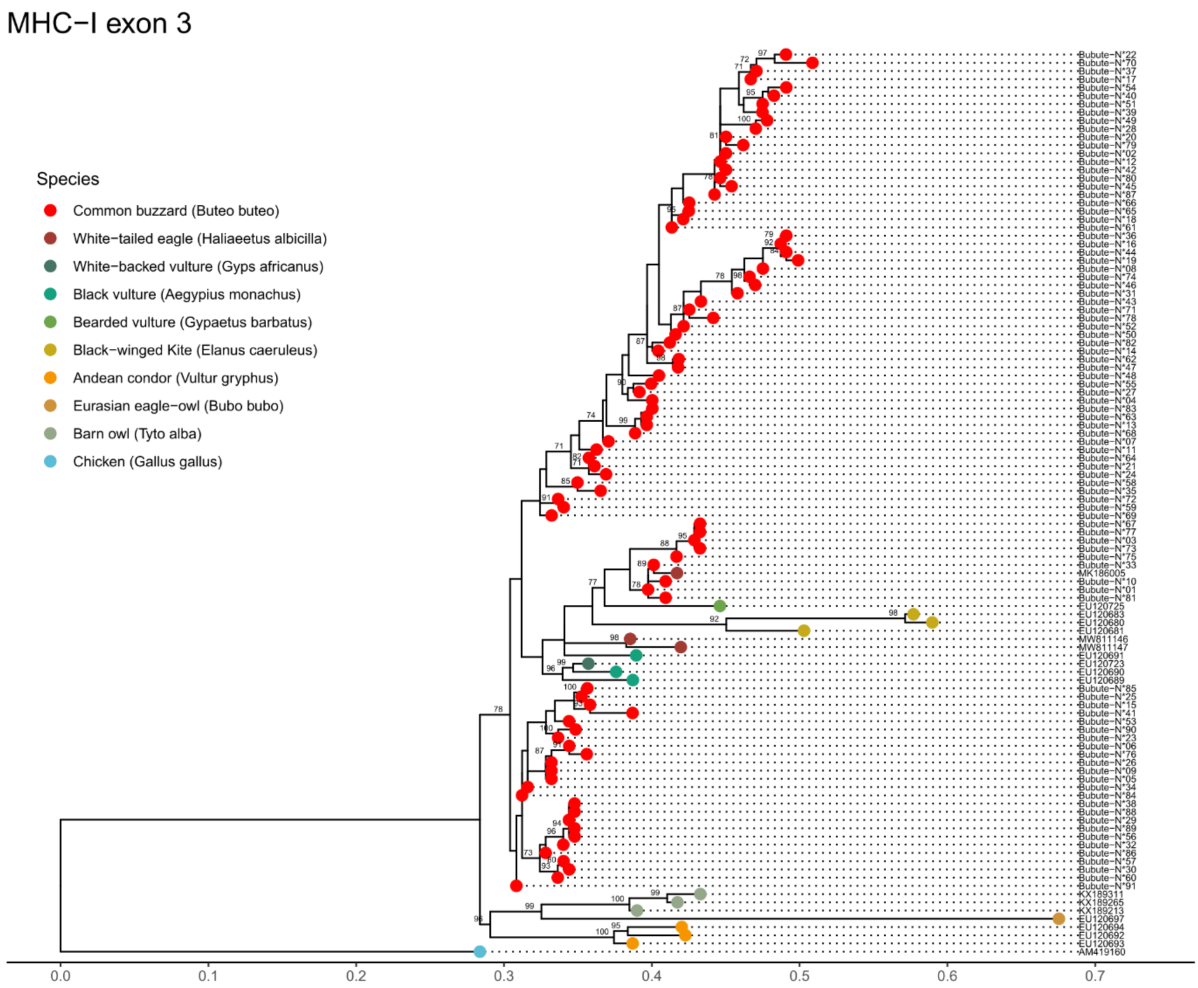

Figure S10. Phylogenetic relationships of common buzzard MHC-IIB exon 2 alleles across closely related Afroaves species from ML-based phylogenies.

Maximum-likelihood phylogenetic reconstruction for 41 *Buteo buteo* alleles of MHC class IIB exon 2 was performed using IQ-TREE [11]. We used ModelFinder [12] implemented within IQ-TREE web server [13] to select the best substitution model based on BIC. Branch support was assessed using 1000 ultrafast bootstrap replicates (UFBoot [14]), using the K2P+R3 model. Scale bars indicate the number of substitutions per site. Values of clades with > 70 bootstrap support are shown. As with Figure S8, alleles do not fall neatly into three clades, as would be expected for three locus copies that evolved independently and for long enough to differentiate from each other. Common buzzard MHC-IIB exon 2 alleles clustered both within and between species, including distantly related species separated 35.1 myr, the white-backed vulture (*Gyps africanus*) and black vulture (*Aegypius monachus*).

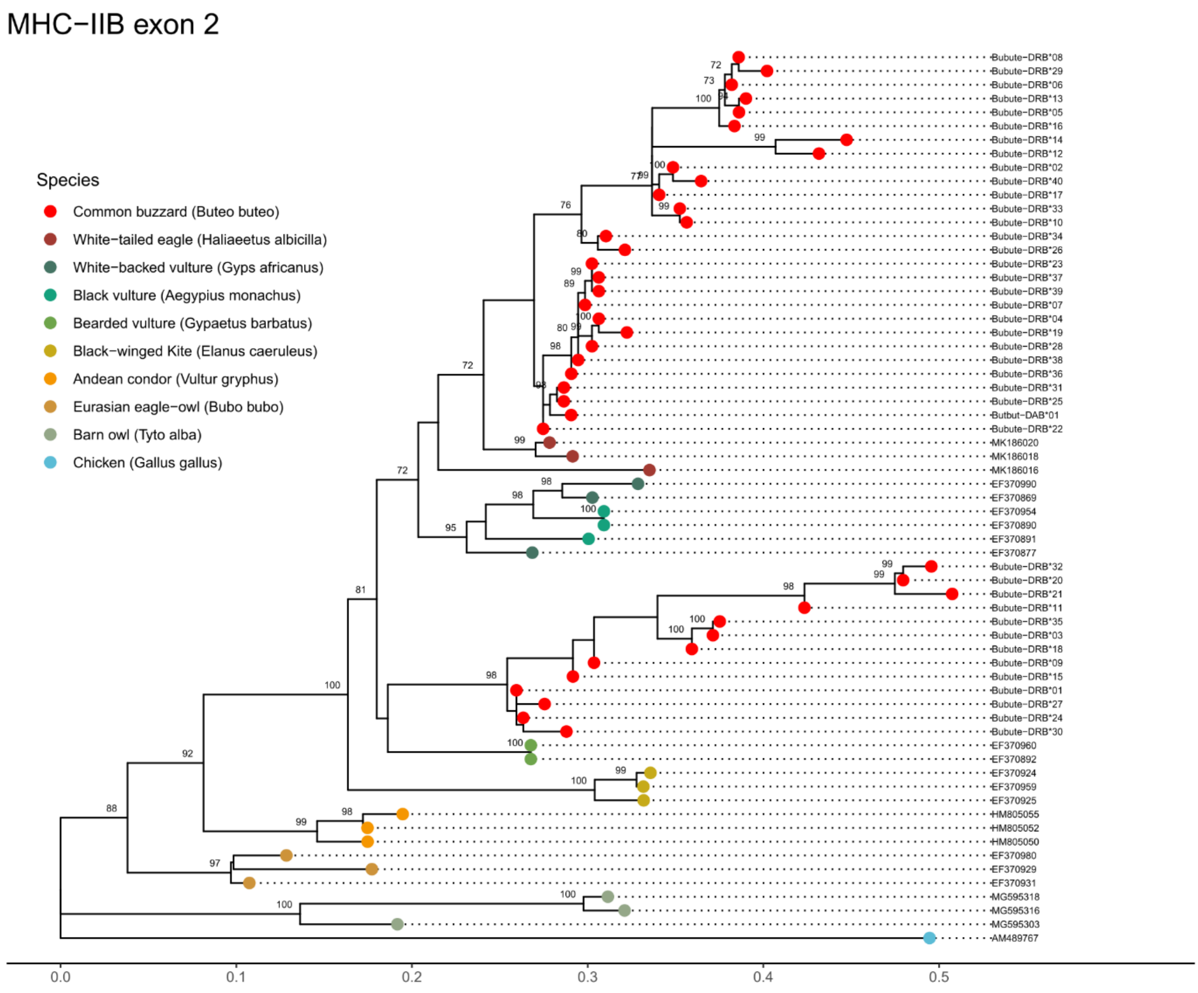

Figure S11. Phylogenetic relationships of common buzzard MHC-I exon 2 and MHC-IIA exon 2 alleles among other avian orders from ML-based phylogenies.

Maximum-likelihood phylogenetic reconstruction for *Buteo buteo* alleles of MHC class I and II was performed using IQ-TREE [11]. We used ModelFinder [12] implemented within IQ-TREE web server [13] to select the best substitution model based on BIC. Branch support was assessed using 1000 ultrafast bootstrap replicates (UFBoot [14]), using the HKY+F+G4 model for MHC-I exon 2 (a) and TIM3e+G4 for MHC-IIA exon 2 (b). Scale bars indicate the number of substitutions per site. Values of clades with > 70 bootstrap support are shown. See Methods for accension numbers. a) As shown for the approximately-maximum-likelihood phylogenetic trees in Figure 4, common buzzard MHC class I exon 2 alleles cluster with Accipitriformes. Vertical red bars show the locus location of the alleles based on haplotype-aware long-read contigs. MHC-I appears to have an ancient Locus 1 lineage shared within the order Accipitriformes with golden eagle (*Aquila chrysaetos*), bald eagle (*Haliaeetus leucocephalus*) and northern goshawk (*Accipiter gentilis*). b) Repeating the patterns of Figure 4, MHC class IIA exon 2 alleles tend to cluster by isotype (such as mammalian DRA and DQA and avian DBA) instead of order. Common buzzard alleles cluster with Accipitriformes DRA isotype and by species with very short branch lengths. Together this suggests common buzzards share a DRA/DAA isotype with other species, and recent duplications occurred in common buzzards.

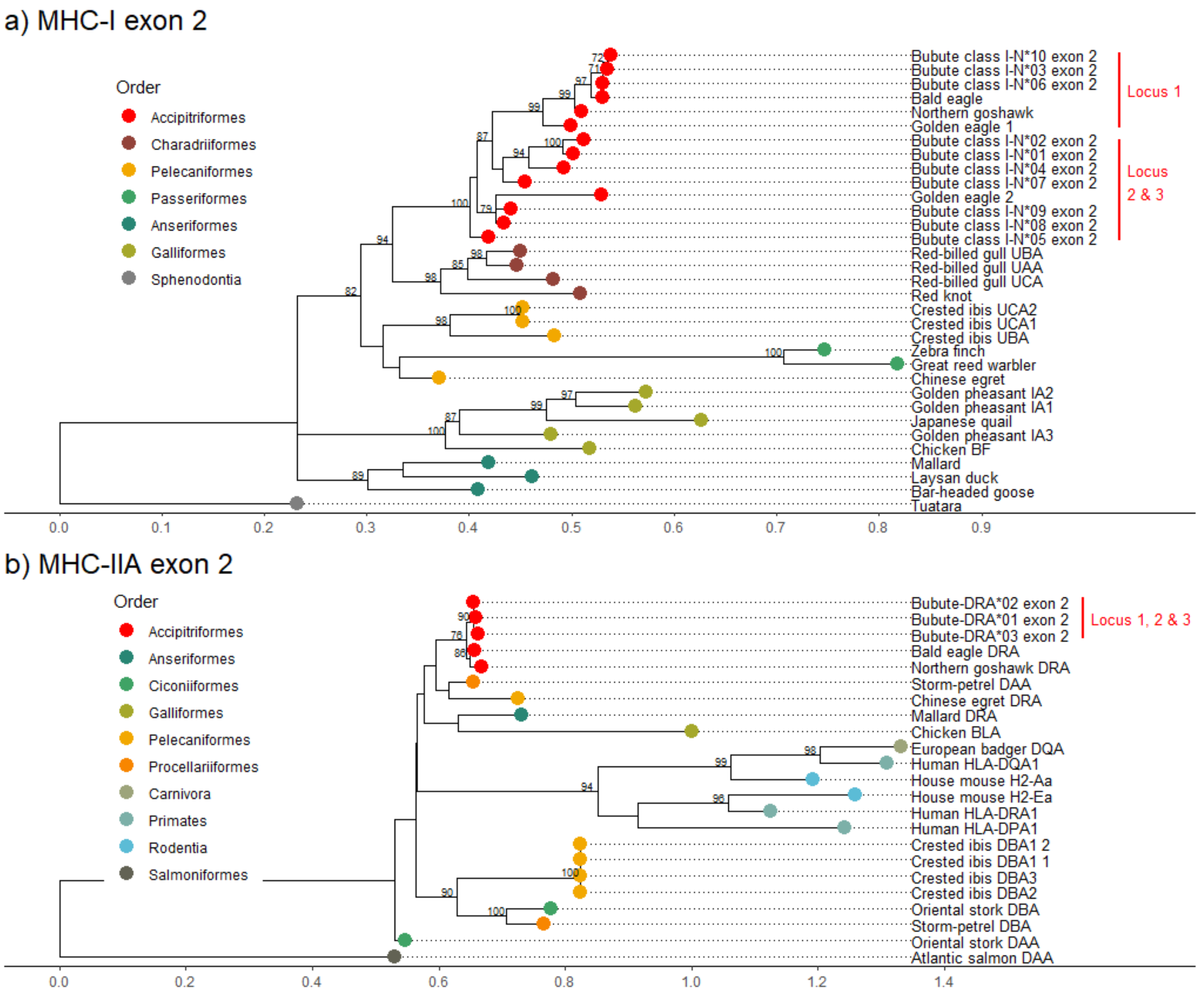

References

1. Martin DP, Murrell B, Golden M, Khoosal A, Muhire B: **RDP4: Detection and analysis of recombination patterns in virus genomes**. *Virus evolution* 2015, **1**(1).
2. Luo X, Kang X, Schönhuth A: **phasebook: haplotype-aware de novo assembly of diploid genomes from long reads**. *Genome biology* 2021, **22**(1):1-26.
3. Li H: **Minimap2: pairwise alignment for nucleotide sequences**. *Bioinformatics* 2018, **34**(18):3094-3100.
4. Hurley CK, Kempenich J, Wadsworth K, Sauter J, Hofmann JA, Schefzyk D, Schmidt AH, Galarza P, Cardozo MBR, Dudkiewicz M *et al*: **Common, intermediate and well-documented HLA alleles in world populations: CIWD version 3.0.0**. *HLA* 2020, **95**(6):516-531.
5. Robinson J, Barker DJ, Georgiou X, Cooper MA, Flicek P, Marsh SG: **IPD-IMGT/HLA Database**. *Nucleic Acids Res* 2020, **48**(D1):D948-D955.
6. Kaufman J, Salomonsen J, Flajnik M: **Evolutionary conservation of MHC class I and class II molecules—different yet the same**. *Semin Immunol* 1994, **6**(6):411-424.
7. Reche PA, Reinherz EL: **Sequence Variability Analysis of Human Class I and Class II MHC Molecules: Functional and Structural Correlates of Amino Acid Polymorphisms**. *J Mol Biol* 2003, **331**(3):623-641.
8. Saper MA, Bjorkman PJ, Wiley DC: **Refined structure of the human histocompatibility antigen HLA-A2 at 2.6 Å resolution**. *J Mol Biol* 1991, **219**(2):277-319.
9. Chappell PE, Meziane EK, Harrison M, Magiera Ł, Hermann C, Mears L, Wrobel AG, Durant C, Nielsen LL, Buus S: **Expression levels of MHC class I molecules are inversely correlated with promiscuity of peptide binding**. *elife* 2015, **4**:e05345.
10. Salomonsen J, Marston D, Avila D, Bumstead N, Johansson B, Juul-Madsen H, Olesen GD, Riegert P, Skjødt K, Vainio O: **The properties of the single chicken MHC classical class II α chain (B-LA) gene indicate an ancient origin for the DR/E-like isotype of class II molecules**. *Immunogenetics* 2003, **55**(9):605-614.
11. Nguyen L-T, Schmidt HA, von Haeseler A, Minh BQ: **IQ-TREE: A Fast and Effective Stochastic Algorithm for Estimating Maximum-Likelihood Phylogenies**. *Mol Biol Evol* 2014, **32**(1):268-274.
12. Kalyaanamoorthy S, Minh BQ, Wong TKF, von Haeseler A, Jermiin LS: **ModelFinder: fast model selection for accurate phylogenetic estimates**. *Nat Methods* 2017, **14**(6):587-589.
13. Trifinopoulos J, Nguyen L-T, von Haeseler A, Minh BQ: **W-IQ-TREE: a fast online phylogenetic tool for maximum likelihood analysis**. *Nucleic Acids Res* 2016, **44**(W1):W232-W235.
14. Hoang DT, Chernomor O, von Haeseler A, Minh BQ, Vinh LS: **UFBoot2: Improving the Ultrafast Bootstrap Approximation**. *Mol Biol Evol* 2017, **35**(2):518-522.
